# Global areas of low human impact (‘Low Impact Areas’) and fragmentation of the natural world

**DOI:** 10.1038/s41598-019-50558-6

**Published:** 2019-10-02

**Authors:** Andrew P. Jacobson, Jason Riggio, Alexander M. Tait, Jonathan E. M. Baillie

**Affiliations:** 1 National Geographic Society, Washington D.C., 20036 USA; 2 Department of Environment and Sustainability, Catawba College, Salisbury, NC 28144 USA; 3 Nicholas School of the Environment, Box 90328, Duke University, Durham, NC 27708 USA; 4 Department of Wildlife, Fish and Conservation Biology, University of California, Davis, California 95616 USA

**Keywords:** Conservation biology, Environmental impact

## Abstract

Habitat loss and fragmentation due to human activities is the leading cause of the loss of biodiversity and ecosystem services. Protected areas are the primary response to this challenge and are the cornerstone of biodiversity conservation efforts. Roughly 15% of land is currently protected although there is momentum to dramatically raise protected area targets towards 50%. But, how much land remains in a natural state? We answer this critical question by using open-access, frequently updated data sets on terrestrial human impacts to create a new categorical map of global human influence (‘Low Impact Areas’) at a 1 km^2^ resolution. We found that 56% of the terrestrial surface, minus permanent ice and snow, currently has low human impact. This suggests that increased protected area targets could be met in areas minimally impacted by people, although there is substantial variation across ecoregions and biomes. While habitat loss is well documented, habitat fragmentation and differences in fragmentation rates between biomes has received little attention. Low Impact Areas uniquely enabled us to calculate global fragmentation rates across biomes, and we compared these to an idealized globe with no human-caused fragmentation. The land in Low Impact Areas is heavily fragmented, compromised by reduced patch size and core area, and exposed to edge effects. Tropical dry forests and temperate grasslands are the world’s most impacted biomes. We demonstrate that when habitat fragmentation is considered in addition to habitat loss, the world’s species, ecosystems and associated services are in worse condition than previously reported.

## Introduction

Habitat loss and fragmentation due to human activities are the leading causes of today’s biodiversity crisis^1,2^. Habitat loss consists of the destruction of natural systems by human actions and is an undisputed principal driver of the loss of biodiversity and ecosystem services^3^. Fragmentation, the subdivision of habitat into smaller and more isolated patches, often occurs in the real world in tandem with habitat loss. Preserving protected areas and remaining intact landscapes are the primary means to address habitat loss and fragmentation, and they are the foundation of resiliency for our changing planet^4,5^. In recognition of their importance, the 2010 Convention on Biological Diversity Aichi Biodiversity target 11 set an achievable goal of 17% of the terrestrial surface and inland waters protected by 2020. Approximately 15% of the terrestrial surface is currently protected^6^. However, recent studies suggest protection levels should be higher to ensure biodiversity, climate and ecosystem services remain at needed levels^7^. There is momentum to increase the targets to 30% protected by 2030 and 50% by 2050^7–10^. Dinerstein *et al*.^11^ recently proposed a Global Deal for Nature, setting out priorities and a template for ways to reach 30% protection levels. Attendees at the upcoming 2020 Conference of Parties (COP-15) to the Convention on Biological Diversity, will decide on targets for how much of the planet should be protected in the future. Therefore, to help determine if these ambitious goals are feasible and where to focus conservation efforts, this study provides a new method to identify the spatial distribution of global human impacts and measures how much of the planet remains in areas not heavily impacted by humans today.

A variety of previous efforts have attempted to discern the global distribution of human impacts, ranging from land cover^12^ to wilderness mapping^13,14^, and from binary to index-based efforts^15^. The efforts most relevant to identifying areas of low human impact are the Human Footprint Index (HFP)^16,17^, Anthromes^18,19^, and Global Human Modification (GHM)^20^. The HFP index combined geographic data on terrestrial human impacts to provide a cumulative index of human pressure. The index varies from 0 to 50, low to high human pressure. HFP was first produced in 2002^16^ and later updated to provide changes in pressure from 1993 to 2009^17^. Anthromes, or anthropogenic biomes, proposed that human and biological systems be analysed together and merged potential natural vegetation cover with classes representing various types of human activity and levels of intensity^18^. Human population and land use land cover data combine with vegetation data to create more than 15 categories (e.g., Urban, Residential Irrigated Cropland, Residential Rangelands). Ellis and colleagues^19^ have also subsequently created time-series data. Most recently, Kennedy and colleagues^20^ quantified the degree of land modification by humans using 13 anthropogenic stressors. The method multiplies the proportion of each grid cell covered by a stressor by an intensity value based on standardized measures of human-induced impacts. The continuous measure of human modification varies from 0-1, low to high modification, approximating the percentage of each cell that has been modified by humans. HFP and GHM have a resolution of 1 km^2^ while Anthromes is ~5 km^2^. Every process has strengths and weaknesses in their methods and spatial output, and we believe the critical question of how much of the world remains in a natural state benefits from a diversity of rigorous, independent and comparable mapping approaches.

Here, we develop a new categorical data set delimiting humanity’s current impact on the terrestrial environment, which we call Earth’s remaining Low human Impact Areas (hereafter Low Impact Areas or LIAs). We use recent, highly-resolved and publicly-available global data on human impacts including human population, livestock density, forest change, land cover and nighttime lights. These data vary in their date of release, spatial resolution and consequence for the environment, but are the most contemporary global data sets of human impact available. We combine these data in a categorical process to identify Earth’s LIAs. LIAs are defined as landscapes that currently have low human density and impacts and are not primarily managed for human needs (e.g., agricultural production, logging, etc.). These are areas where natural processes predominate, but are not necessarily places with intact natural vegetation, ecosystem processes, or faunal assemblages. We map these regions at 1 km^2^ resolution to identify ecoregional and global patterns and proportions of LIAs. Importantly, this global approach is repeatable and allows tracking of LIAs through time, and the data set matches the highest resolution and most contemporary data sets available on global human impacts. Furthermore, LIAs provide a transparent perspective to mapping the spatial extent and intensity of global human impacts.

Our process was explicitly created to enable us to go a step beyond calculating rates of habitat loss to also investigate habitat fragmentation. Fragmentation can lead to widespread, long-term changes in the composition and function of remaining habitat^21^. While there is an ongoing debate about the impacts of fragmentation (the altered spatial configuration of habitat for a given amount of habitat loss) *per se*^22^, recent research has reiterated the negative consequences of fragmentation itself^23^. Habitat loss is closely monitored, and in some ecosystems is tracked daily (e.g., Global Forest Watch GLAD alerts). In contrast, fragmentation is not explicitly tracked and is less well understood^21^. While many attempts have been made to estimate global habitat loss^1,12,24^, there is but a single estimate comparing fragmentation across biomes^20^, and none comparing against an historic baseline. We identify human-derived changes in habitat fragmentation, by comparing LIA patches to an idealized globe with no human-caused fragmentation at a global scale and on a biome-by-biome basis. These findings demonstrate that when habitat fragmentation is considered in addition to habitat loss, the world’s species, ecosystems and associated services are in much worse condition than previously reported.

## Results

### Low impact areas

**Figure 1:**
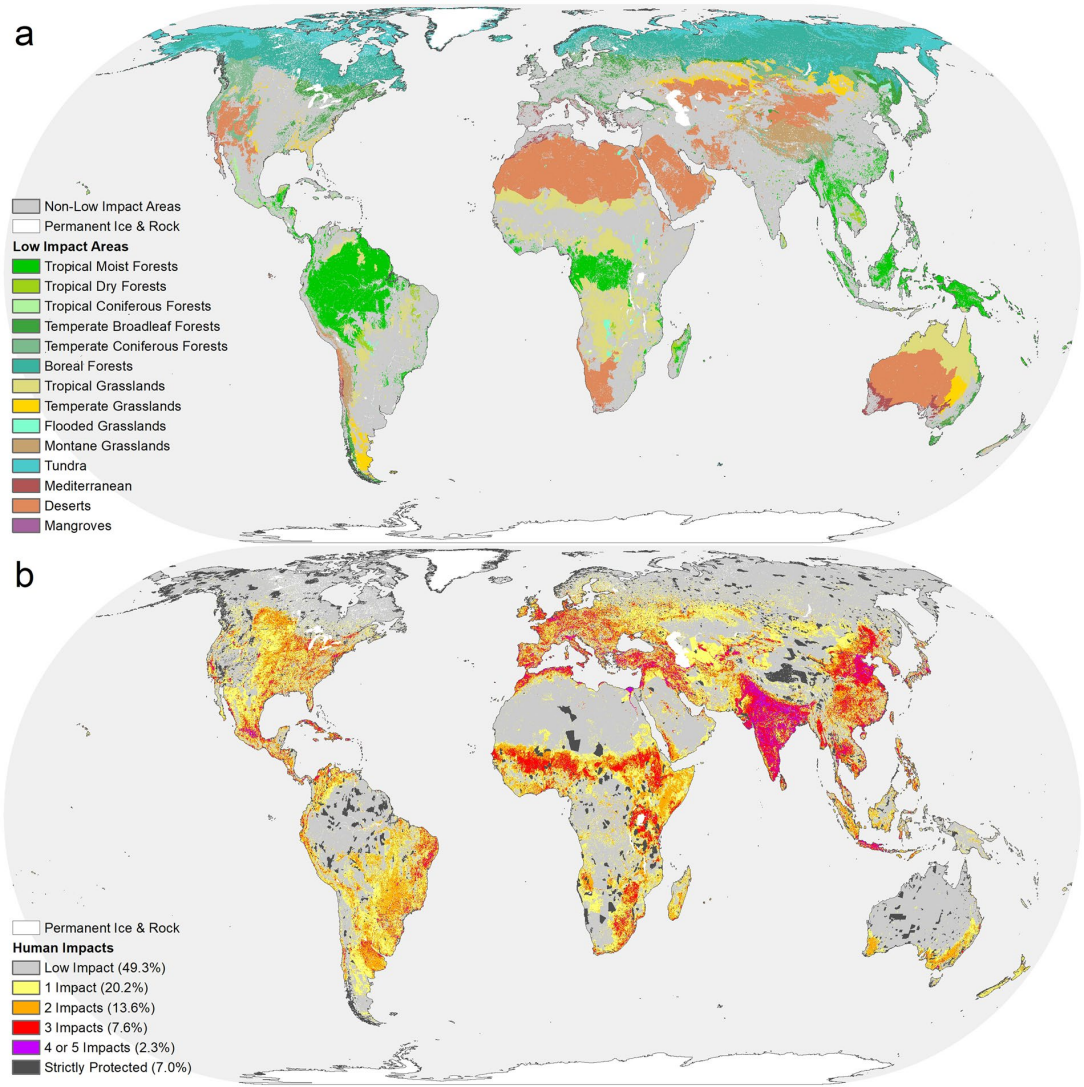
(**a**) Global map of Low Impact Areas (color-coded by biome) and the surrounding human-dominated matrix in gray. (**b**) Global map of the intensity of human impacts. The majority of impacted cells have more than one impact.

**Figure 2:**
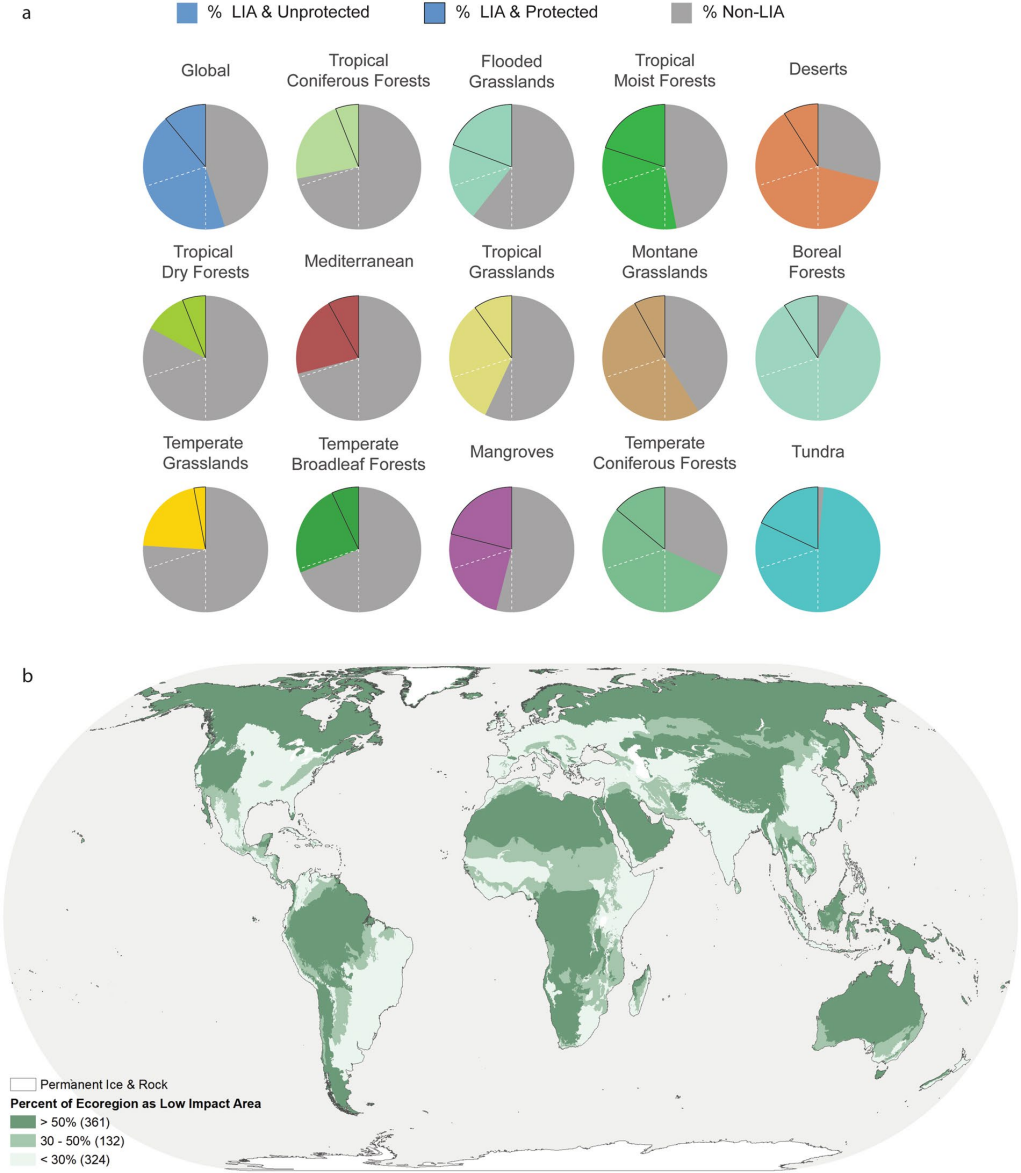
(**a**) Pie charts showing the percent of Low Impact Areas and protected areas (all categories I–VI) per biome with dashed lines showing 30% and 50% protection targets. Biomes are ordered top-to-bottom, then left-to-right in order of increasing amount of Low Impact Area. (**b**) Global map highlighting ecoregions that have between 30% and 50% Low Impact Area.

We found just over half (56%) of the planet is in Low Impact Areas distributed non-randomly across all continents and biomes (Fig. 1). Tropical dry forests and temperate grasslands were the two most extensively converted biomes on the planet, each with less than a quarter of their extent as LIA (Fig. 2). (Note that we use abbreviated names for the biomes; see Supplementary Table S1 for the full names). Tropical coniferous forests, Mediterranean and Temperate broadleaf forest biomes each had ~30% of land remaining as LIA (Table 1). Three additional biomes, flooded grasslands, tropical grasslands and mangroves each had less than 50% of land remaining as LIA. Tundra and boreal forests had the highest remaining percentage of LIA, both greater than 90%.

**Table 1:** Key habitat loss and fragmentation statistics by biome. Percent increase and decrease are changes from an idealized globe with no anthropogenic fragmentation.

Biome	Baseline area (km^2^)	% Low Impact Area	% Low Impact & protected*	% Increase in patch #	% Decrease in mean patch size	% Decrease in core area	Median distance to non-LIA edge (km)	% Decrease in median distance to edge
Globe	128,876,330	56.2	10.7	1254	95.8	53.4	6.0	—
Tropical dry forests	3,828,365	16.8	5.7	1979	99.2	89.6	1.6	93.8
Temperate grasslands	10,427,615	24.3	2.7	6165	99.6	84.3	2.2	97.2
Tropical coniferous forests	681,771	28.3	6.0	988	97.4	82.5	1.6	76.7
Mediterranean	3,258,904	29.2	8.8	2742	99.0	81.0	2.1	93.5
Temperate broadleaf forests	12,219,667	30.6	6.6	2406	98.8	83.6	1.4	97.6
Flooded grasslands	1,099,701	36.8	16.1	466	93.5	72.3	3.0	84.4
Tropical grasslands	21,295,437	43.0	10.4	3254	98.7	65.7	4.3	95.9
Mangroves	271,220	45.8	21.0	91	76.0	70.8	2.2	49.3
Tropical broadleaf forests	19,297,433	53.0	19.9	1973	97.4	56.7	3.9	93.8
Montane grasslands	4,745,258	58.7	7.4	474	89.8	52.5	4.0	89.0
Temperate coniferous forests	3,687,134	68.1	13.9	1379	95.4	49.5	2.6	87.8
Deserts	26,247,964	70.6	8.8	2077	96.8	34.2	11.6	90.0
Boreal forests	14,359,339	92.4	9.3	331	78.5	18.7	5.6	95.2
Tundra	7,456,521	99.3	17.6	5	5.6	1.9	31.8	48.1

^*^Protected includes all IUCN protection categories I–VI.

Similar to biomes, ecoregions varied widely in terms of the remaining proportion of LIA (Fig. 2). Even within a single biome, some ecoregions could be nearly 100% LIA while others were nearly 0% (Supplementary Fig. S1, Table S2). While four of 14 biomes had less than 30% of land remaining as LIA, this was true for 323 (39%) of ecoregions. Tropical dry forests and temperate grasslands biomes had the highest percentage of ecoregions not meeting a 30% LIA threshold at 78.2% and 66.0% respectively. Eight biomes had less than 50% of land remaining as LIA, and 455 (55%) of ecoregions did not meet this target. Tropical dry forests and Mediterranean biomes had the highest percentage of ecoregions not meeting a 50% LIA threshold, at 94.5% and 85.0% respectively.

Not just the extent but also the intensity of human impact varied widely. Multiple human impacts affected the majority (54%) of areas not identified as LIA (Fig. 1). Of the world, 7% was both in LIAs and covered by strictly protected areas (IUCN protection categories 1-4), an additional 4% was covered by other protected areas, and the remaining 45% was in LIAs only.

### Validation

We used the open-access Human Footprint validation data set composed of ~3000 1 km^2^ validation plots^25^. Of 3009 plots, Venter and colleagues^17,25^ identified 1752 with low human pressure and 1257 with high pressure based on visual inspection of recent, high-resolution satellite data. We found 78.5% of low-pressure validation plots were in LIAs and 76.8% of high-pressure plots were outside LIAs (Supplementary Fig. S2, Table S3). This resulted in a Cohen’s kappa statistic of 0.55.

### Global fragmentation

We applied our data set of low human-impacted areas to assess the level of fragmentation at global and regional scales using both patch-based statistics and Euclidian distance to edge. As biomes vary naturally in their inherent level of fragmentation, we calculated a baseline level of fragmentation and the difference from this baseline to assess the impact of humans. We identified the baseline layer using the current demarcation of land and water^26^, intersected this with biome distributions^9^, and assumed no human impact. We then compared the baseline to LIAs.

The baseline terrestrial land area was composed of 73,374 non-ice-or-snow-covered fragments ≥1 km^2^ in size (Supplementary Table S4). In contrast, we found 993,656 LIA fragments globally, representing a 1254% increase in patch number. While the number of tundra fragments was essentially the same (only a 5% difference), temperate and tropical grasslands had the highest percentage increase in number of patches, at 6165% and 3254% respectively (Table 1).

**Figure 3:**
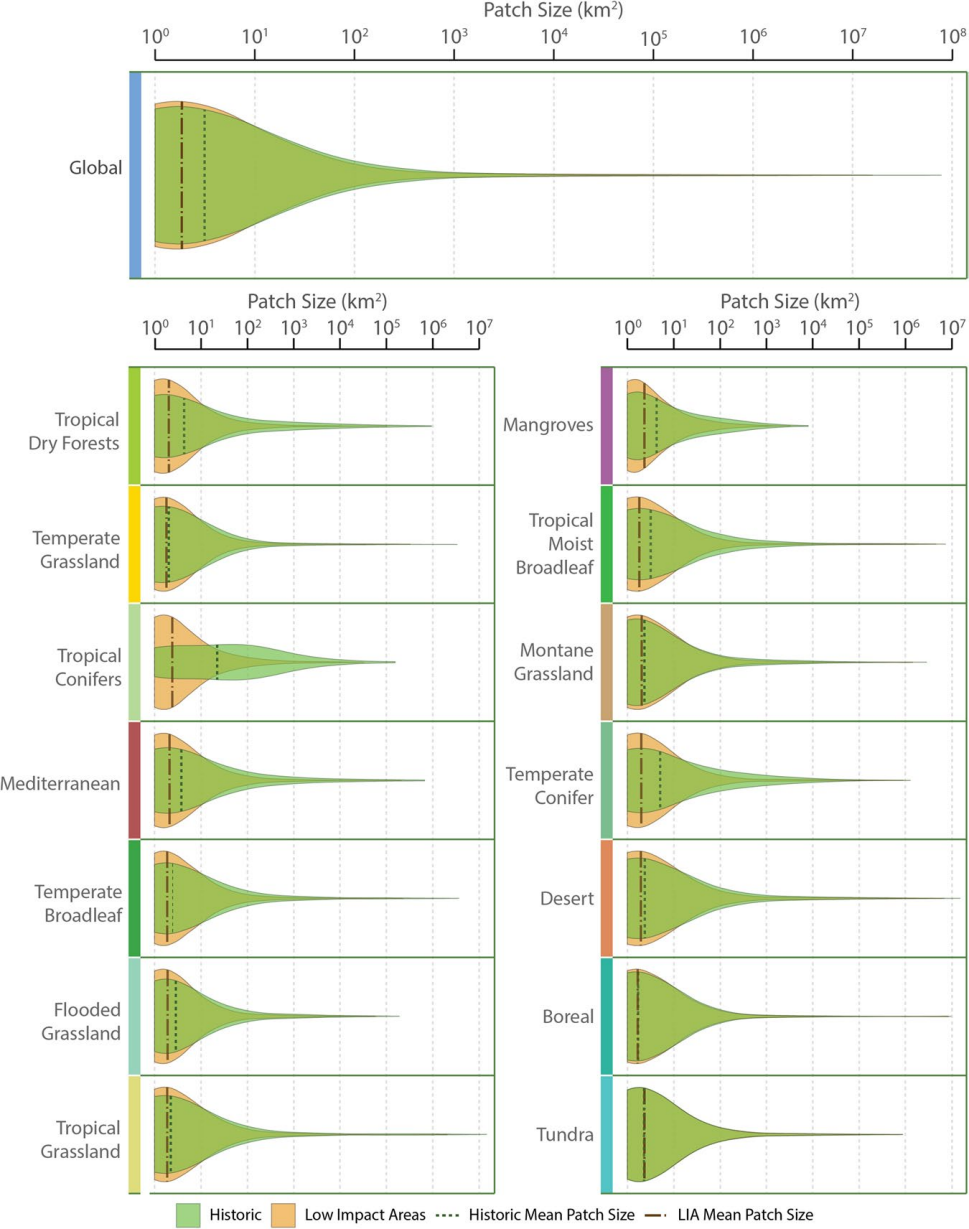
Patch size distributions across biomes in the baseline and current Low Impact Areas. Violin plots show the probability density of the data at different values. The wider the plot, the more common the patch size. These highlight the change in the proportion of patch sizes between baseline and current Low Impact Areas, such as the loss of medium-sized patches and gain in frequency of the smallest patches in Low Impact Areas. Biomes are ordered top-to-bottom, left-to-right in order of increasing percent Low Impact Area. Figure designed by TerraCommunications.

**Figure 4:**
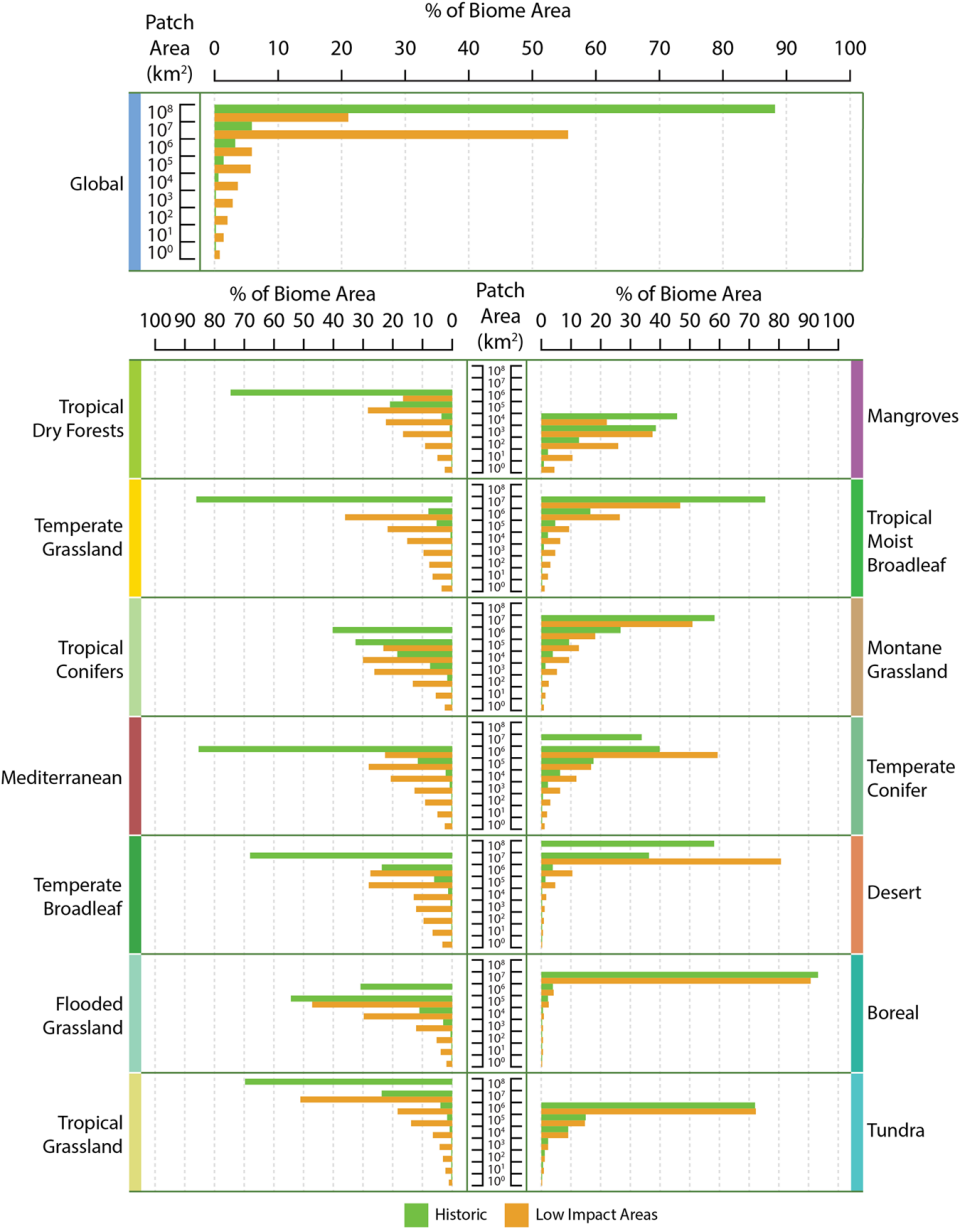
Histograms of total contributed area from all patches within that patch size bin between the baseline and current Low Impact Areas. Several very large patches dominated baseline distributions of most biomes. But, with the exception of the boreal and tundra biomes, the largest patches are splintered and the amount of area found in small and medium-sized patches is substantially greater in Low Impact Areas than the baseline. Biomes are ordered top-to-bottom, then left-to-right in order of increasing percent Low Impact Area.

Fragmentation involves the splintering of large and medium-sized patches into a proliferating number of smaller ones. At the global scale, and in nearly all biomes, the proportion of large and medium-sized patches decreased, while the smallest patches, those between 1 and 10 km^2^ in size, increased (Fig. 3). All biomes with the exceptions of tundra and boreal forest exhibited an increase in the proportion of small-sized patches relative to the overall number of patches. As the largest patches have decreased in size, not only are small fragments more common, the smallest fragments also represent a greater proportion of overall area than they did (Fig. 4).

Concomitant with an increase in patch number and an increasing proportion of small patches, average patch size has decreased substantially (Table 1). Globally, average patch size decreased by 95%. Indeed, in 11 of 14 biomes, average patch size declined by over 90%, with temperate grasslands and tropical dry forests suffering the highest decreases of over 99%. Most biomes, across baseline and current LIAs, had a median patch size of 1 km^2^.

Smaller patch size also leads to decreases in core area. Using a 1 km buffer to define edge vs. core (e.g.^21,27^), global core area has declined by 53%. The pattern of core area loss mirrored the pattern of overall impact with tropical dry forests and temperate grasslands experiencing the greatest percent loss in core area at 90 and 84% respectively.

**Figure 5:**
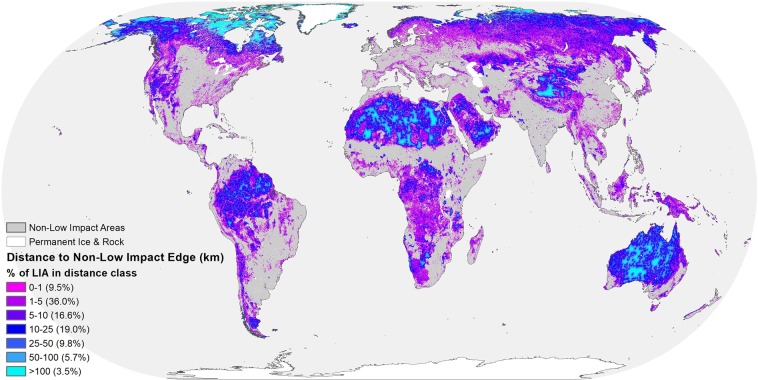
Global distance to non-low impact edge. The shape and size of Low Impact Areas contribute to varying distances to non-Low Impact Area edge cell. Low Impact Areas furthest from non-low impact edge cells are in the Arctic, the Sahara Desert, the Australian Outback, and the Tibetan Plateau.

However, core area represents only one aspect of distance to edge; distance to edge varies continuously within a patch (Fig. 5). Globally, the median distance to a non-LIA edge cell was only 6 km (Table 1). Several biomes including tropical dry forests, tropical coniferous forests, and mangroves have a median distance to edge of less than 2 km.

Yet, some biomes are naturally more fragmented (i.e. mangroves), so we also identified the percent decrease in median distance to edge between the baseline and LIAs. Distance to edge here included both distance to biome edge (including ocean coastline) and to non-LIA cell to isolate the impact of human pressures. Temperate broadleaf forests had the largest decrease in median distance to edge of all biomes between the baseline and LIA, decreasing from 58 km to 1.4 km, or by 97.7% (Table 1).

## Discussion

We present a new categorical data set of human impact on the planet, Low Impact Areas, to provide an additional assessment on the feasibility of meeting expanded protected area targets on land minimally impacted by people. We used a transparent process, with the most current, open-access, publicly-available data sets to assess current human impacts across the entire terrestrial surface of the Earth not permanently covered by ice, snow, or water. This process identified 56% of land in landscapes that currently have low human density and impacts, not primarily managed for human use. The LIAs are heavily fragmented and increasingly exposed to edge effects. While tropical dry forests had the lowest remaining percentage of LIA of all biomes, temperate grasslands exhibited the greatest fragmentation rates.

We find slightly over half of land is in LIAs. This is similar to other data sets suggesting roughly half of land is in areas minimally impacted by people^9,20,28^. Although Oakleaf and colleagues^24^ found 76% of land as “natural,” Watson and colleagues^29^ only found 23% as ‘wilderness.’ This variation demonstrates how direct comparisons across data sets of human impact are difficult as definitions are critical and vary by publication (i.e., ‘natural,’ ‘wild,’ ‘impacted’ etc.), and often there are differences in the extent of analysis (e.g., Oakleaf and colleagues included permanent rock and ice areas in the extent). The most relevant comparisons to our process include the Human Footprint Index^17^ Anthromes^19^, and Global Human Modification^20^. The HFP was thresholded, finding roughly 40% of the terrestrial surface is “no” or “low pressure”^17^. The Anthromes data set identifies ~45% of the world as wild or semi-natural^19^. Kennedy and colleagues^20^ map 49% of land as having low modification. See Supplementary Text 1 (including Supplementary Table S5, Figs S3 and S4) for a more detailed comparison of methods and output between LIA, Anthromes, HFP and GHM.

Similar to Anthromes, we used a categorical process to determine whether a 1 km^2^ grid cell currently has low human impacts. If a grid cell had any urban or cropland extent, nighttime lights, or forest cover change (with minor exceptions—see Methods), then it was no longer low-impact. The impacts of both human population and livestock density vary with ecosystem productivity. All things equal, a more productive environment can support more livestock or people than an area the same size in a less productive environment^30^. Thus, in hyper-arid regions we set the threshold for impact to one person or livestock unit per km^2^. Impacts were then scaled by aridity; such that higher densities were required to move a cell from low impact to non-low impact in more humid environments (but see the section on Sensitivity in Supplementary Materials).

An advantage of this transparent process is that it allows identification of which and how many inputs caused a particular place to be identified as impacted. Roughly, 20% of all land experienced only one human impact layer, while two or more layers impacted 24%. This has implications for restoration, suggesting that less than half of non-LIA land has only one type of human impact affecting it. A further advantage of the process is that input data sets are regularly updated, in some cases yearly, and hence LIAs can be easily tracked over time.

To assess our accuracy, we used the 3114 × 1 km^2^ open-access Human Footprint validation plots. These sample areas have a median image resolution within plots of 0.5 m, and median image date of 2010. LIA validation metrics were comparable to those of the HFP. Venter and colleagues^17^ found 78.8% agreement of low impacted validation points in areas found to have low HFP scores (compared to our 78.5% agreement in LIAs). They also found 76.1% agreement of high-pressure areas in areas with high HFP scores (compared to our 76.3% agreement in non-LIAs). This suggests that our product performs similarly to a thresholded version of the HFP (index values 0,1,2), despite our data set predicting ~14% more of the terrestrial surface of the planet as being low human impact than the HFP. Therefore, we felt confident in applying the data set to identify global fragmentation rates.

Fragmentation, along with habitat loss, are together considered the primary reasons behind biodiversity loss and associated declines in ecosystem function^21^. While there is widespread agreement of the large and pernicious effects of habitat loss on biodiversity, there is less agreement on the impact of habitat fragmentation *per se*^22,24^. We do not reiterate the statements on either side of this debate but instead present the global fragmentation results side-by-side with a measure of habitat loss to review the conservation status of different biomes.

Biomes vary naturally in their baseline level of fragmentation, with some more contiguous than others. Mangroves, for instance, are spread patchily along tropical coastlines with no very large patches at the global scale. Tundra and boreal forest are widespread biomes dominated by large patches. The tropical coniferous forests biome exists in only a few areas of the globe, and uniquely had more patches at mid-sizes than the smallest-sized patches in the baseline scenario. When comparing fragmentation rates across biomes, it is important to first consider the natural level of fragmentation in that biome, to properly assess the impact of human-caused fragmentation.

Regardless of the previous distribution of patch sizes, humanity has had a homogenizing effect across the biomes. Now, all biomes have similar distributions of patch sizes in LIAs. This result matches the homogenization of the size distributions of tropical forest fragments across continents found by Taubert *et al*.^31^. Indeed, we speculate that the homogenization of patch sizes across biomes is a factor in overall biotic homogenization^32,33^. Both globally, and in most biomes, large patches dominated total patch area in the baseline scenario. While still predominantly true, the current largest patches are frequently an order of magnitude smaller than the baseline, and the percentage of total area made up by the largest patches has shrunk considerably (e.g., from 97% to 50% in tropical coniferous forests; Supplementary Table S4). Much more of the remaining habitat is in small and medium-sized patches which are exposed to edge effects with less core area. All biomes have at least 88% of total patches as small patches in LIAs whereas the smallest baseline percentage was 40% (Supplementary Table S4).

Despite this similarity across biomes, there is wide variation across biomes in the level of fragmentation caused by humans. Temperate grasslands have suffered the worst anthropogenic fragmentation of all biomes. Average patch size has decreased by over 99%, core area has decreased by 84%, and the number of habitat patches has increased by 6265%. Median distance to edge has also decreased by 97.2%. Tropical dry forests, tropical grasslands, Mediterranean and temperate broadleaf forest biomes have also experienced substantial decreases in average patch size, core area, increases in patch number, and decreases in median distance to edge. With so little habitat conversion, it is unsurprising that the tundra biome has experienced the least anthropogenic fragmentation to date, although climate change threatens to rapidly modify this biome regardless of direct human impact.

At the global scale, humanity has caused widespread fragmentation of terrestrial environments. Globally, average patch size has decreased by 95.8%, core area has decreased by 53.4%, and the number of habitat patches has increased by 1254% from a baseline with no human-caused fragmentation. Furthermore, median distance to edge metrics highlight the increasing exposure of remaining LIA to human impacts, with the median global distance to edge of only 6 km.

Most previous analyses looked only at fragmentation rates within a single biome or region (e.g., forests^21,31,34^; North America^35^). Haddad and colleagues^21^ found 70% of all forested areas are within 1 km of an edge, which is substantially higher than our estimate of 27% when aggregating across all forested biomes. Haddad and colleagues^21^ likely overestimated the amount of edge as they used a threshold of 30% tree cover to determine if a cell was forested or not, hence creating an edge anywhere forest cover dipped below this threshold. Our estimate is an underestimate as we do not include edges caused by roads. When roads were included (in identifying ‘Very Low Impact Areas’ – see below), the proportion of forest within 1 km of an edge rose to ~60%. Recently, the GHM layer was used to assess global fragmentation rates across all biomes, although they only measured distance to edge. Across all biomes, we find a smaller median distance to edge than Kennedy *et al*.^20^, in part as we included distance to ocean (a biome edge). We also found differences between ranking of biomes by threat. Kennedy *et al*.^20^ identified tropical coniferous forests, tropical dry forests, mangroves and flooded grasslands as the most fragmented biomes. While we also identified tropical dry forests as one of the most fragmented biomes, the other three biomes Kennedy mentioned have three of the four smallest baseline mean patch sizes by biome, suggesting that some of the fragmentation they identify is not anthropogenic in origin.

We caution that our data set of human impacts has several caveats. One caveat is that the delineation of LIAs is dependent on the use or exclusion of various data sets, such as roads (see the section on Sensitivity in Supplementary Materials). Another caveat is that we set all impacts equal to each other and there is no accumulation of impacts. We recognize that the impact of livestock on biodiversity is not equal to that from conversion to cropland^36^ (again, see the section on Sensitivity in Supplementary Materials). Additional caveats to our process are true of other global human impact mapping efforts such as GHM and HFP. Some areas with low human impacts currently (e.g., Scotland) have experienced greater human impacts in the past and their current environmental status and biodiversity are substantially altered from a prior baseline. We also recognize that some human impacts are not readily available in existing data sets, such as global hunting rates or invasive species’ distributions. Thus, we do not suggest LIAs as places that still unequivocally contain intact native flora and fauna. But, it is likely that the only areas that do have intact native species assemblages are contained within LIAs. In addition, we do not assess remaining natural intact vegetation, merely the absence of identifiable human stressors. For example, within the mangrove biome, neither GHM, HFP or our method assesses if mangrove trees are still present. (Another complication for mangroves is that they exist in small patches on the coastline and due to variation in coastline delineation across data sets, some were ruled as NoData – see Methods.)

There are also a number of caveats to our fragmentation findings. First, these numbers are dependent on the baseline land and biome data we use as well as the scale of the analysis. The baseline fragments are a result of the intersection of biome data and terrestrial land after the removal of water and permanent snow or ice. A different baseline land or water data set, along with a finer analysis scale, would find more patches. Indeed, a recent analysis suggested the median tropical forest fragment was <0.001 km^2^ ^31^. Another important caveat is that we treat all edges the same, regardless of biome or the context of the edge (see the section on Sensitivity in Supplementary Materials). Most other regional or global studies treated all edges the same^20,21,27^, but Pfeifer and colleagues^37^ created a novel way to look at the intensity of the edge, or the contrast, between cells with varying amounts of forest cover. In addition, what constitutes an edge differs between biomes (e.g., woody encroachment into or desertification of a grassland). Despite these caveats, we believe this analysis makes an important contribution towards assessing global rates of fragmentation across all biomes. Future, more nuanced, global assessments of fragmentation could vary the definition of edge by ecosystems.

In conclusion, tropical dry forests and temperate grasslands stand out as the two biomes with the smallest remaining percentage of land remaining as LIAs and the greatest increases in fragmentation rates. This confirms results that also show these biomes as some of the most threatened globally^12,20^. As early as 1988, there were warnings that tropical dry forests were highly threatened^38^. Miles and colleagues^39^ also made a broad call for conservation of this biome. This biome does boast a relatively high rate of protection within LIAs; however, dry forests are still under severe threat (e.g., the Gran Chaco is a global deforestation hotspot)^40^. Temperate grasslands are also recognized as a biome with a very favourable climate for human activity and this suitability has led to large-scale habitat conversion. Despite the longstanding recognition that temperate grasslands are highly threatened^41,42^, very little is protected in remaining LIAs. The Mediterranean, temperate broadleaf forest and tropical coniferous forest biomes are clustered together and round out the top five most threatened biomes in our analysis, having both high rates of loss and high levels fragmentation. Tropical grasslands also stand out as having higher levels of anthropogenic fragmentation than expected given their percent remaining in LIA.

Beyond a comparison of the conservation status of various biomes, this analysis finds that 56% of the planet remains in Low Impact Areas. Encouragingly, this supports the feasibility to protect 30% of the planet by 2030 and 50% by 2050 using predominately natural landscapes not primarily managed for human use. We recognize these low human impact areas are not ‘wilderness’ areas with full assemblages of species, and we affirm the importance of slowing the loss of global wilderness^29^ and of biodiverse habitats^43^. Yet, ecosystem services can still be provided by extensive, if not ‘intact’ natural systems^44^. Finally, we recognize that the representativeness target of the protection goals will be more difficult to achieve as particular ecoregions and biomes are already heavily impacted and inhabited (Supplementary Table S2)^9,11^. Restoration, along with expanded protected areas, will be needed to achieve global protection and representation goals^45^.

## Materials and Methods

### Process for establishing LIAs

In identifying potential input data sets to identify LIAs, we examined the literature for spatially explicit data sets on human impacts, including those used by HFP^16,17^, Anthromes^18,19^, and GHM^20^. We used five criteria to select appropriate inputs: (1) directly relevant to mapping human impacts on the environment, (2) open access, (3) global in extent without substantial data gaps (i.e., every country needed data), (4) the most recently updated version of the data set, and (5) 1 km^2^ raster cell size or finer. For all identified input data sets but land cover and human population density, this left a single potential input data set. For land cover and population density, we selected those data sets with yearly time series to allow for future updates. A few data sets used to identify other human impacts were not included in our process as they did not fit one or more of our five criteria (e.g. mines are not mapped globally). In addition, we chose not to use any data set on human accessibility (but see^46^) as this was duplicative (both human population density and accessibility are modeled using the location of settlements and transportation infrastructure).

To map LIAs, we followed a categorical process that started with the entire globe as low impact and then excised areas that are primarily managed or modified for human use (Supplementary Fig. S5, Table S6). First, we used the Ecoregions 2017 layer to establish terrestrial area and eliminate oceans^9^. Then, we excluded water bodies, and permanent snow and ice using the ESA Climate Change Initiative Land Cover 2.0 data set for the year 2015^26^ by reclassifying these areas as NoData. Also using the ESA land cover data set, we removed all areas classified as cropland, regardless of the portion present in the cell (Values = 10, 11, 12, 20, 30 or 42), and urban (i.e., primarily managed for humans). Similarly, we removed cells containing nighttime lights using the nighttime VIIRS day/night band composites^47^. To do this, we used the 2015 annual vcm-orm-ntl with outliers removed and background (non-lights) set to zero. Any cell with a positive radiance value was deemed anthropogenic in nature and no longer low-impact.

Next, we excised areas using human population and livestock density data. Human population data were obtained from the LandScan High Resolution Global Human Population Data Set^48^ for the year 2015, and the livestock density layer was derived from the Gridded Livestock of the World v.2 for the year 2006^49^. We combined livestock (goat, sheep and cattle) into a single scaled Tropical Livestock Unit^50^. Neither LIAs, or wilderness^29^, explicitly exclude people or livestock, yet their impacts vary with ecosystem productivity. A more productive environment can support more livestock or people than a similarly-sized area in a less productive environment^30^. In addition, there is no scientifically agreed upon number of people or livestock that a region can contain before being considered “highly impacted” or “managed primarily for humans”. Therefore, we rescaled these density data sets with the Global Aridity Index^51^, using a multiplication factor of 1 for humid regions, 2 for dry sub-humid landscapes, 4 for semi-arid areas, 8 for arid regions, and 16 for hyper-arid landscapes. We then reclassified the modified human population and livestock data sets such that values greater than 16 were classified as non-low impact and values less than 16 were classified as low impact. In this system, to exceed the threshold for ‘impact’, a human population density of >16 was needed in humid regions, whereas only a human density of >1 was needed in hyper-arid regions (see the section on Sensitivity in Supplementary Materials to examine the impact of this choice). We performed this process separately for each density data set.

Then, we removed forest cover change as a potential form of human management of landscapes. We split the world into two zones by biome (as delineated by 9): subtropic and tropic zone (including flooded grasslands and mangroves), and all others. The only difference between these two zones was the application of data on fire extent. We downloaded MODIS collection 6, MCD64A1 Burned Area extent globally from the start of collection, November 2000, through December 2015 using the AppEEARS and the DAAC2Disk tool of LPDAAC^52^. We merged the data across all years to create a global burned area extent raster to match forest cover change data from 2000–2015 (Global Forest Watch)^53^. In the non-tropic-or-subtropic zone, burned area extent often matched forest loss and growth extent indicating natural forest loss and re-growth due to wildfires^54^. Although some fires in this region are anthropogenic in origin, we assumed they were not precursors to agriculture or settlement, e.g., land cover change. The two data sets were slightly misaligned and so a small buffer of ~1.5 km was set around burned areas to better encompass forest change from fire. Forest loss could occur the year of a fire, or within a few years following a fire as a lower intensity fires may not kill trees outright, and hence data were summed and compared across all years rather than matched year-by-year. All forest cover change from within the buffered burned area extent was deemed natural. However, fire is often used as a tool to clear land in subtropical and tropical forests^55^, therefore we did not use burned area extent to modify forest cover change data in these areas. In sum, we removed all forest loss and forest gain from potential LIAs, minus buffered burned area extent in the boreal/temperate zone, assuming that loss was most likely due to anthropogenic reasons such as forestry practices or land conversion^54^, and gain was primarily due to conversion to agroforestry or plantation (e.g., oil palm, rubber, *Eucalyptus spp*., *Pinus taeda*).

Finally, we added back in certain protected areas that are managed primarily for biodiversity conservation (i.e., not primarily managed for humans) to correct for some known errors of the input data layers. For instance, the Masai Mara National Reserve in Kenya is well protected from habitat conversion, although the land cover data set suggests it is wall-to-wall cropland. Thus, we added in protected areas with IUCN Categories I–IV (strictly protected areas) that are currently designated at the national level using the World Database on Protected Areas^6^ along with some country-specific data sets including: national nature reserves from China^56^, formal A protected areas from South Africa^57^, national parks, game reserves and nature reserves from Tanzania^58^, and protected areas in the United States with Gap Analysis Program Status 1 or 2 (“managed primarily for biodiversity”)^59^. While some protected areas are indeed “paper parks”; globally, strictly protected areas have far lower levels of human pressure than those with more permissive uses^60^ and have higher species richness and abundance^61^ than adjacent unprotected lands.

We projected all data to the Eckert IV equal-area projection, resampling to 1 km^2^ cells from their native spatial resolution using the nearest-neighbor method (30 arc-seconds for the majority of input data; Supplementary Table S6). All geospatial analyses were conducted in ArcGIS Desktop 10.5 and ArcGIS Pro (Esri, Redlands, CA).

### LIA metrics

We ran several summary statistics on the resulting LIAs. We calculated the number and size of LIAs, and calculated the overall extent of the world remaining as low impact, and on a biome and ecoregion basis. We also identified the overlap between LIAs and protected areas as identified in the data sets above.

### Validation

We conducted validation using an existing open-access global validation data set from the Human Footprint project^25^. Each plot was visually scored for signs of human impact. All validation points were visually scored according to the level of various human land cover and activities seen within the 1 km^2^ plot. Background satellite imagery varied in resolution and date, but the median image resolution was 0.5 meters with median acquisition year of 2010. We only used the 3114 sample plots assessed with high confidence and, of these, an additional 105 were located in NoData regions of our data set (primarily permanent ice and snow) and excluded. We used the same threshold as Venter and colleagues^17^, a visual score of one, to identify areas of human impact. We compared the 3009 plots with LIAs to assess accuracy. We applied Cohen’s kappa statistic, a measure of agreement between two categorical data sets that accounts for expected agreement by chance, to measure accuracy.

### Fragmentation

To assess the impact of anthropogenic fragmentation on the natural system, we first needed a baseline level of fragmentation. We calculated fragmentation statistics on both the baseline data and current LIAs, and compared them to identify the impact of human-caused fragmentation on biomes. We intersected LIAs with biome data^9^ to identify current levels of habitat fragmentation per biome. A biome boundary may split an otherwise contiguous LIA into two (or more) patches. The number and size of patches were calculated per biome. We also calculated core area using a 1 km buffer from edge (e.g.^21,27^) using Fragstats 4.2^62^. When calculating global fragmentation rates, we did not intersect the background land or LIA data sets with biome distribution and hence have slightly different area totals. Finally, we calculated Euclidean distance to edge in two ways. The first, distance to edge, looked at every cell within a LIA and calculated its distance to an ocean or non-LIA cell. Results were intersected with biome boundaries to identify the median distance to edge per biome. The second method accounts for the natural fragmentation differences between biomes. For instance, mangroves are inherently patchy, and even if the entire mangrove patch remained LIA but areas just outside it are modified, distance to edge values are smaller than other biomes as distance to the edge of the biome is small. Therefore, just like the other statistics, we calculated distance to edge on both the baseline data and after intersecting LIAs with biomes to get a change metric. In this baseline scenario, the distance to edge was simply distance to biome edge. To calculate distance to edge for LIAs, distance could be either to non-LIA cell, or the biome/ocean edge. These two values were then compared to identify change. The Euclidian Distance to Edge tool in ArcGIS converts every cell to a centroid before calculating the distance between them, so adjacent cells ideally have a distance of 1 km (binned in the 0–1 km class), while diagonally-adjacent cells have a distance of 1.4 km. In both instances, we calculate distances after removing rock, ice and inland water such that distances accumulate across these cells but are not counted in the calculation. We used the geodesic calculation method which identifies the shortest distance between two points on the earth’s surface on a spheroid, hence accounting for the curvature of the earth, and eliminating distortion effects from geographic projections. This method is more accurate than previous global fragmentation analyses which did not account for projection or distance measurement methods. This method was performed in ArcGIS Pro 10.2.

The baseline land was set as the minimum land area between all input data, minus water and permanent snow and ice, overlaid with updated 2017 biome distributions^9^. Rock and ice from Dinerstein and colleagues^9^ were retained as ‘tundra’, while permanent snow and ice (along with water) from the ESA land cover data set was used as a mask^26^. Coastlines varied between the different input layers (Supplementary Table S6). The final terrestrial land extent was thus the minimum area comparable across all data sets (i.e., a cell became NoData if any data set had missing data there). Land was then intersected with biome information such that each cell of land was associated with a particular biome. As before, coastlines between the biome and land data sets did not match perfectly, and thus the minimum matched area was used. Therefore, an area like Greenland was not considered a single island composed of tundra but rather many fragments of tundra separated by permanent snow and ice.

We recognize that this baseline is an idealized and simplified understanding of fragmentation levels, and we make an important assumption that the baseline landscape was entirely unused primary vegetation or ‘low impact’ in this context (similar to^2^). However, as previous publications have suggested^2,19,63^, humans have modified Earth’s land cover for millennia. We do not use these data (e.g., HYDE) as a baseline because they are modeled data sets with inaccuracies and errors, and it is simpler to use the same base data set as that used for the current time period to facilitate comparison and change from original.

### Sensitivity testing

We explored the impact of the use of geospatial road data sets on LIA creation (See Supplementary Text 2). In this scenario, “Very Low Impact Areas”, we used the same process and data sets, except we included all roads as an additional human impact and did not scale impacts by aridity (i.e., kept a threshold of less than one person or livestock unit per km^2^ across all aridity levels). This scenario resulted in only 34% of the planet in a very low impact state (See Supplementary Text 2 including Supplementary Figs S6, S7, Table S7). The most threatened biomes remained the same as in the LIAs, although there were some differences in fragmentation statistics when comparing biomes. Importantly, tropical moist forest is the only high-biodiversity biome, of the five biomes with some ecoregions having >50% of area in Very Low Impact (Supplementary Fig. S7).

We separately examined the impact of the inclusion of human population and livestock density data sets, and at various scaling factors, on the extent of LIAs and fragmentation (Supplementary Text 3). Specifically, we examined the impact on the extent of LIAs from the inclusion of each human stressor input using a ‘leave-one-out’ analysis (Supplementary Table S8). In addition, we examined the impact of removing human population and livestock density input data sets on fragmentation levels. Using the same process to delineate LIAs but excluding human population or livestock density as stressors, 75% of the world is in a ‘modified’ low impact state. Broadly speaking, the impact on fragmentation results is as expected, with median distance to edge increasing for nearly all biomes, and some biomes with more livestock grazing (e.g., temperate grasslands) more impacted than others (e.g., tundra) (Supplementary Table S9 and Fig. S8). Relatedly, we also examined the impact of various aridity-scaling factors on the delineation of LIAs (Supplementary Table S10). In summary, the results from these tests showed that the identification of LIAs is most sensitive to the inclusion of livestock density data, and the choice of aridity-scaling; however, in terms of the impact on fragmentation, the order of the biomes from shortest to longest median distance to edge is quite stable across the various examples.

Finally, fragmentation rates also depend on contiguity rules. We used a 4-cell adjacency rule (rather than 8-cell) for all analyses as this was more conservative. Hence, cells touching only on the diagonal were regarded as two separate patches. We reviewed how the results changed if using an 8-cell adjacency rule (Supplementary Table S11). Changing from a 4 to an 8-cell adjacency rule led to fewer overall patches in both baseline and LIA settings, but for most biomes, led to a greater percentage increase in patch number between the baseline and LIA. While results did vary across biomes and patch fragmentation statistics, the ranking of biomes by threat was unchanged.

## Supplementary information

Supplemental Information

Supplemental Table S2

Supplemental Table S3

## Data Availability

Original data sources are publicly available as described in text and supplemental material. Low Impact Area GIS layer produced by this paper is available on Dryad (10.5061/dryad.z612jm67g).
