# Supplementary material for: Global areas of low human impact (‘Low Impact Areas’) and fragmentation of the natural world
Source: Sci Rep. 2019 Oct 2;9:14179. doi: 10.1038/s41598-019-50558-6 (PMC6775135; doi:10.1038/s41598-019-50558-6)
Supplement: Supplementary file 1 — Supplemental Information [file 41598_2019_50558_MOESM1_ESM.docx]

**Supplementary Information for Fragmented Earth**

Global areas of low human impact (‘Low Impact Areas’) and fragmentation of the natural world

Andrew P. Jacobson, Jason Riggio, Alex Tait, Jonathan Baillie

**Table S1. Full biome names as per Ecoregions 2017 and our abbreviated names.**

**Full biome name** **Abbreviated name**

Boreal forests/taiga Boreal forests

Deserts & xeric shrublands Deserts

Flooded grasslands & savannas Flooded grasslands

Mangroves Mangroves

Mediterranean forests, woodlands & scrub Mediterranean

Montane grasslands & shrublands Montane grasslands

Temperate broadleaf & mixed forests Temperate broadleaf forests

Temperate conifer forests Temperate coniferous forests

Temperate grasslands, savannas & shrublands Temperate grasslands

Tropical & subtropical coniferous forests Tropical coniferous forests

Tropical & subtropical dry broadleaf forests Tropical dry forests

Tropical & subtropical grasslands, savannas & shrublands Tropical grasslands

Tropical & subtropical moist broadleaf forests Tropical moist forests

Tundra Tundra

*
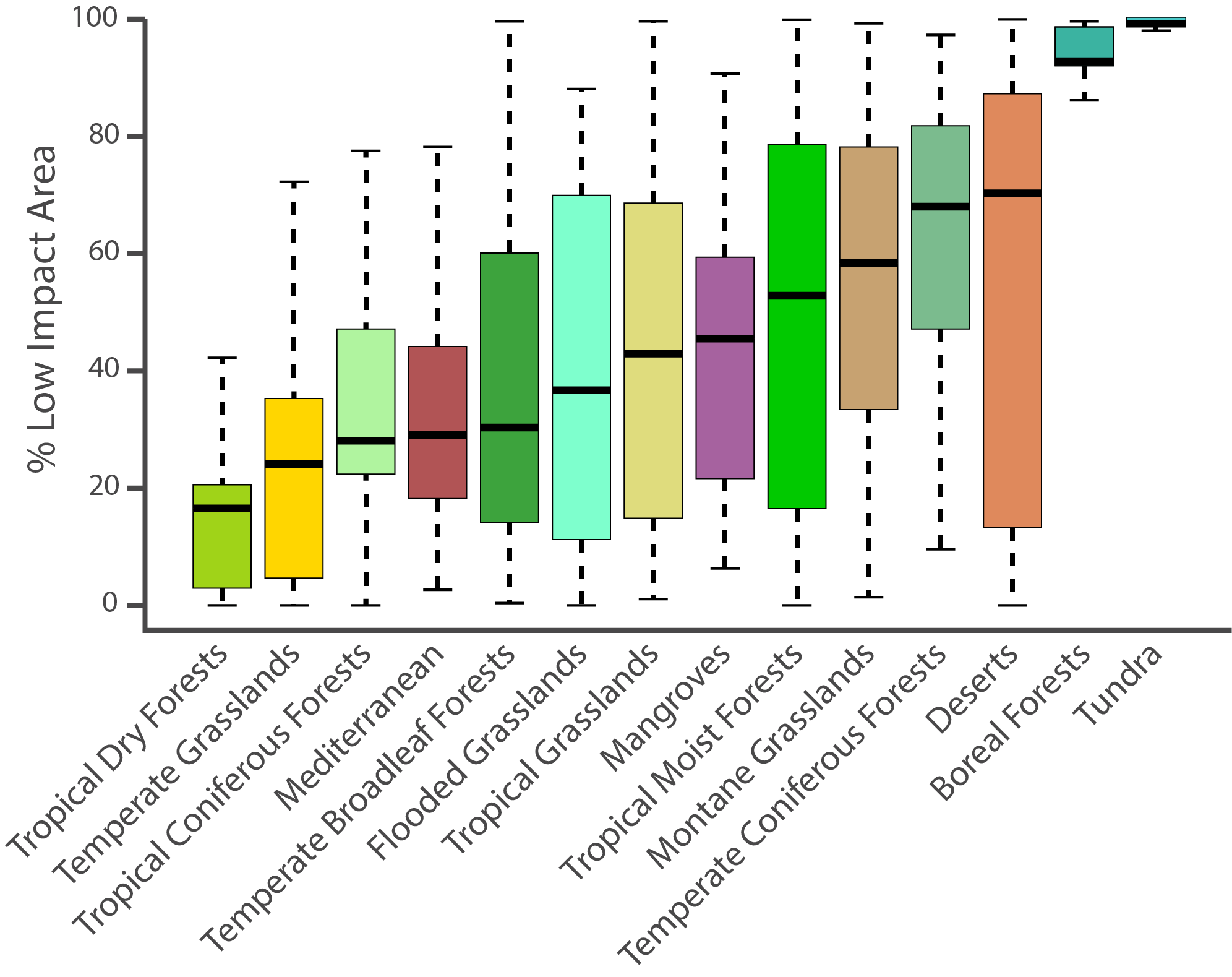
*

**Figure S1.** Percentage of biomes and ecoregions in Low Impact Areas**.** The solid line represents the percent Low Impact Area for the biome as a whole. The boxplot shows the range of percent Low Impact Area calculated for each ecoregion across all ecoregions in a biome. Full ecoregion data are available in Table S2. Biomes are ordered left-to-right in order of increasing proportion of Low Impact Area.

**Table S2.** See separate excel sheet for a list of ecoregions and their percentage in Low Impact Area.


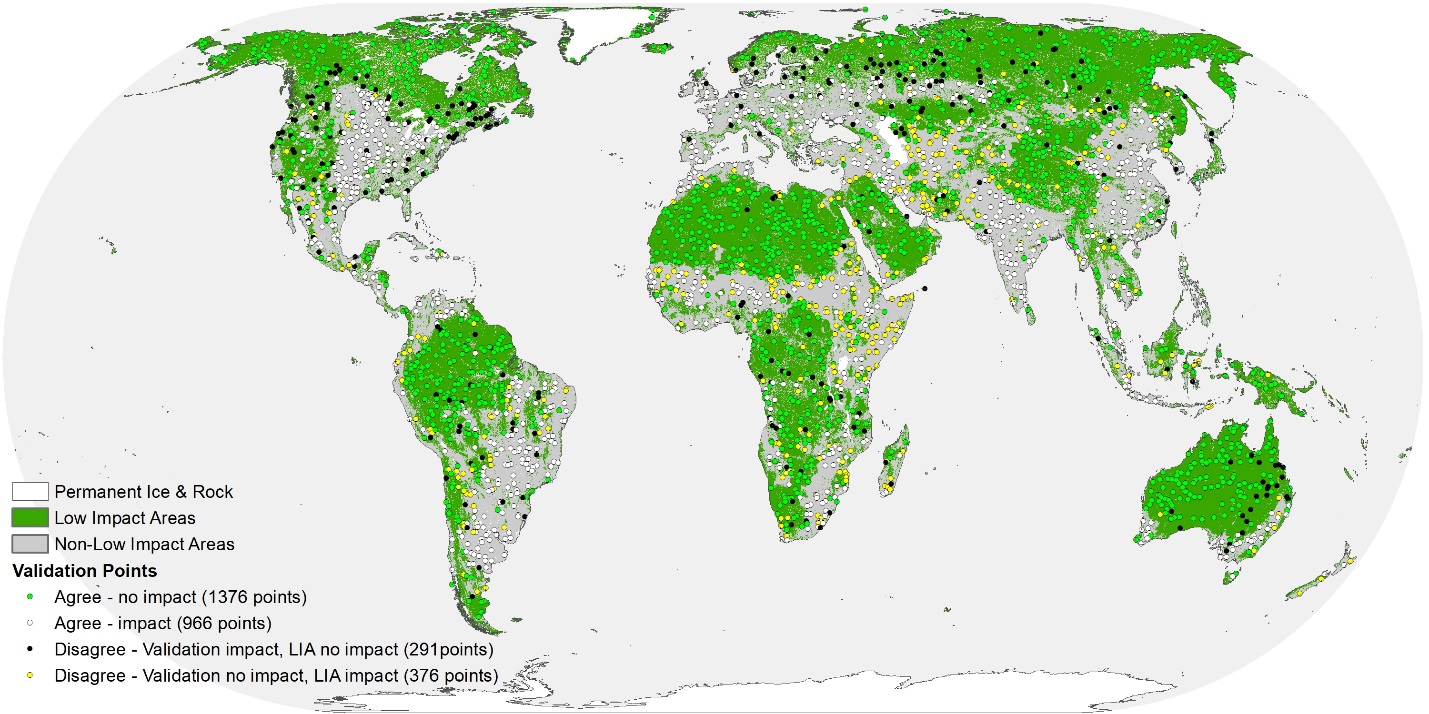
 **Figure S2.** Distribution of Human Footprint validation plots (Venter et al. 2016a) and their classification accuracy.

**Table S3.** See separate excel sheet for more detailed validation results.

**Table S4.** Fragmentation statistics globally and across the 14 biomes.

**Supplementary Text 1 – comparison of Low Impact Areas to existing data sets**

Mapping the distribution and intensity of human impacts on the planet is not a new endeavor. Initial attempts at global human influence mapping started in the 1980s and 1990s (e.g., McCloskey & Spalding 1989, Hannah 1994, UNEP-WCMC 2000). Data and analytical methods have improved substantially since then.

Several recent, well-known methods are similar to our Low Impact Area method including Anthromes (Ellis and Ramankutty 2008; Ellis et al. 2010), Human Footprint Index (Sanderson et al. 2002, Venter et al. 2016b), and most recently, Global Human Modification (Kennedy et al. 2019). While having broadly similar goals of identifying human impacts on the planet, each of these various methods have differences between them and our own. The differences start with the definition of the data set itself, which in turn affects the selection of various human stressor variables and how they are weighted and combined (Table S5). Naturally then, these methodological differences lead to variations in the spatial output.

**Table S5.** Comparison of methods used in creating Low Impact Areas with three recent global human influence data sets.

|  | Anthromes | Global Human Modification | Human Footprint | Low Impact Areas |
| --- | --- | --- | --- | --- |
| Resolution | ~5 km^2^ | 1 km^2^ | 1 km^2^ | 1 km^2^ |
| Data year | 1700 – 2000 by century, and yearly after 2000 | 2015 | 1993 and 2009 | 2015 |
| Type | Categorical | Continuous | Ordinal | Categorical |
| Scaling | 6 groups; 19 classes | 0-1 (Low to high) | 0-50 (Low to high) | 2 classes |
| Definition | Human biomes – “the globally significant ecological patterns created by sustained interactions between humans and ecosystems” | Ecological condition of lands based on the spatial extent and intensity of human activities | Cumulative human pressure on the environment | Landscapes with low human densities and impacts, and not primarily managed for human needs |
| Primary stressor data sets | 6 (Human population density, built-up area, cropland, rice area, irrigated area, pasture) | 13 (human population density, built-up area, cropland, livestock, major roads, minor roads, two-tracks, railroads, mines, oil wells, wind turbines, power lines, night-time lights) | 7 (human population density, cropland, pasture, major roads, railroads, navigable rivers, night-time lights) | 7 (human population density, built-up area, cropland, livestock, forest cover change, roads, night-time lights) |
| Calculation of spatial extent | Classifications based on proportion of total area experiencing the stressor | Determined the proportion of the cell modified by each stressor per 1 km^2^ area (values ranged from 0 to 1) | Treated each stressor layer as binary | Treated each stressor layer as binary |

Each of these data sets are available on their own respective web viewers or FTP-server:

- Anthromes: <http://ecotope.org/anthromes/maps/> and via HYDE <https://themasites.pbl.nl/tridion/en/themasites/hyde/download/index-2.html>,
- Human Footprint Index: <https://wcshumanfootprint.org/>, and
- Global Human Modification: <http://s3.amazonaws.com/DevByDesign-Web/Apps/gHM/index.html> .

To compare with LIAs, we downloaded each of the data sets, including the 2009 HFP and 2015 Anthromes. We projected all data sets to the same Mollweide projection (native for HFP and GHM) and then masked each to the sum of all the data sets. This forced all data sets to have the same boundaries (e.g., ocean coastline), and ultimately the same number of cells with data. Anthromes was down-sampled to have the same resolution (1 km^2^) as the other three data sets. Once all data sets were consistent, we made each data set binary by identifying a threshold value that translates to as close to 56% of the planet (the same that is LIA) as possible. For instance, with Global Human Modification, we start at a value of 0 (or least impact), and 5% of the planet, and gradually raise the value towards 1 (greater impact) until ~56% of the planet is in a ‘lower impact’ bin. This threshold value is 0.13 and splits the planet into two nearly equal halves of 55% and 45%. We repeat this for each of the remaining two comparison data sets. The discrete (or categorical) nature of the data sets means that we cannot obtain a threshold that gives an exact 56/44 split.

We map the three comparison data sets, set to binary thresholds of ~56/44, with LIAs in Figure S3. In addition to the comparison at global extent, we also compare all four data sets at a larger scale (Figure S4).

| 1. Low Impact Areas - 56% | 1. Anthromes 2015 - 53.4% containing 5 categories: Remote Rangelands, Remote Woodlands, Semi-natural Treeless & Barren Lands, Wild Woodlands, and Wild Treeless & Barren |
| --- | --- |
| 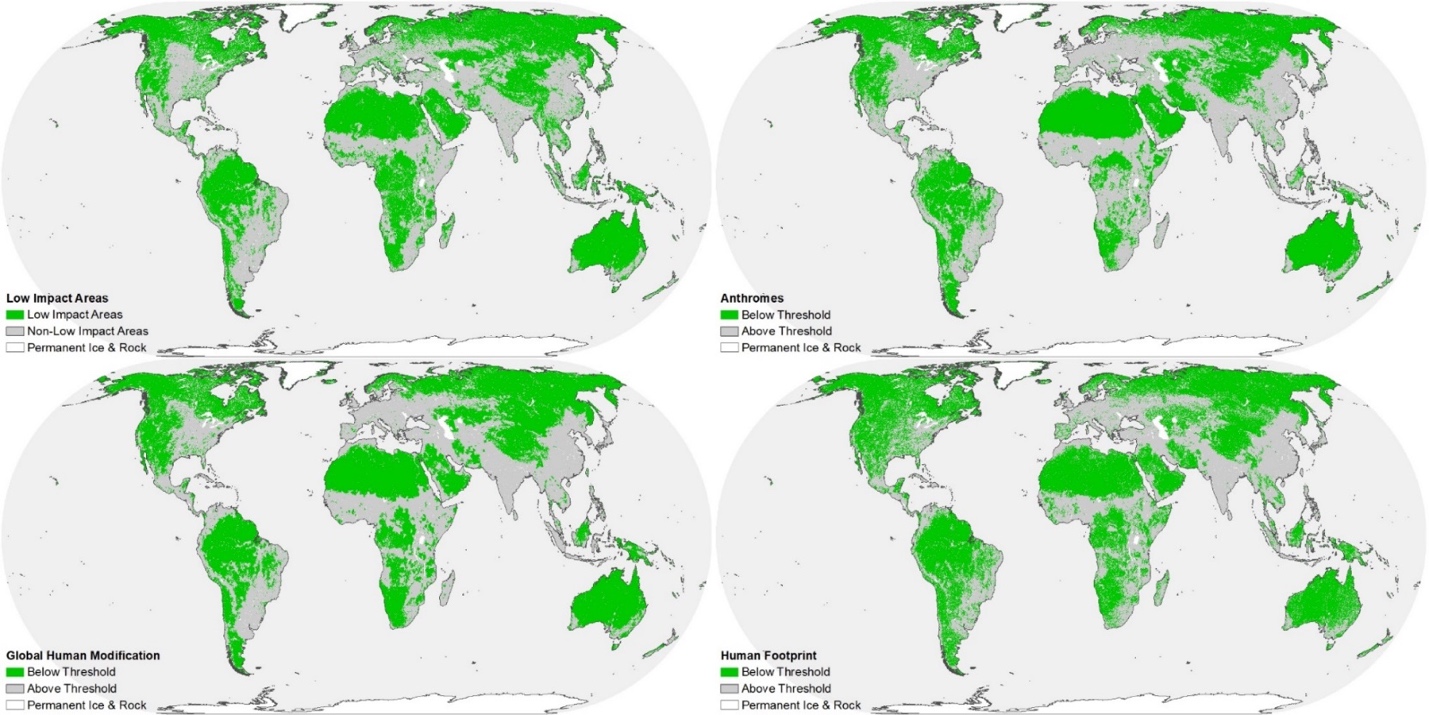 | |
| 1. Global Human Modification - 55.3% below a threshold value of 0.13 | 1. Human Footprint Index 2009 – 56.7% below a threshold value of 4 |

**Figure S3.** Comparison of the four key human impact data sets binned into low and non-low impact regions representing roughly 56/44% of the planet.

| 1. Low Impact Areas | 1. Anthromes 2015 |
| --- | --- |
| 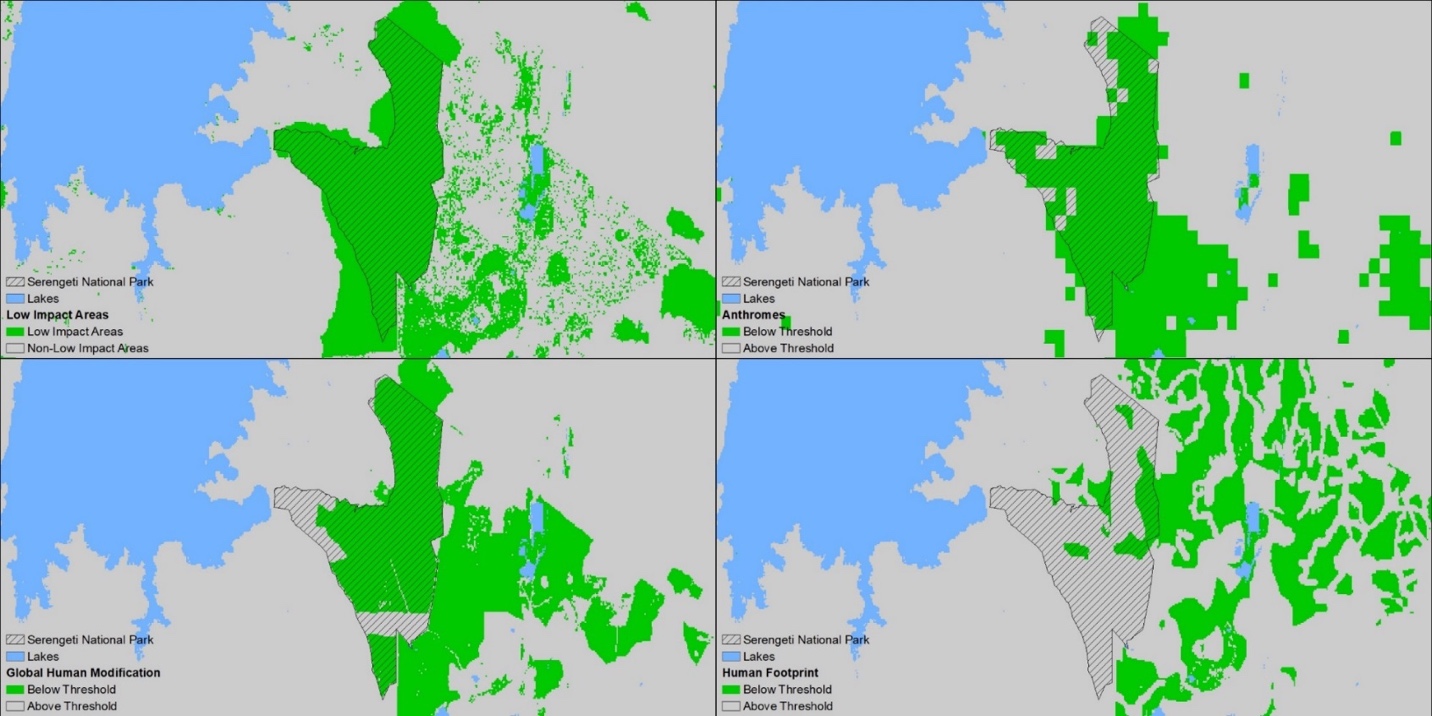 | |
| 1. Global Human Modification | 1. Human Footprint Index 2009 |

**Figure S4.** Large-scale map centered on Serengeti National Park, Tanzania (transparent hashed polygon), comparing the four data sets binned into low and non-low impact regions.


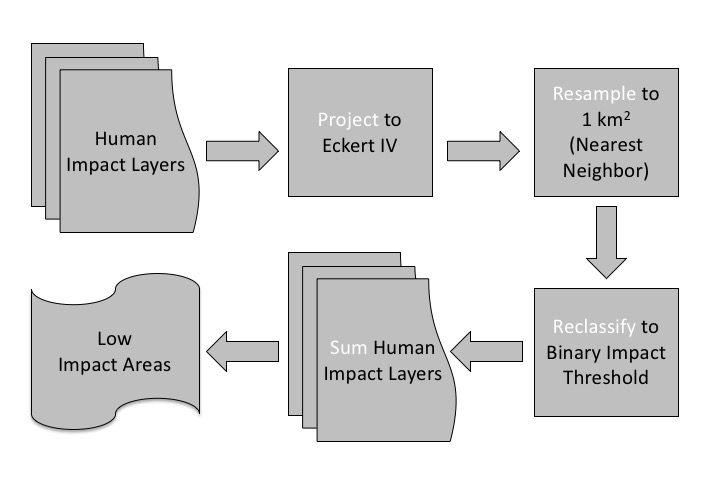


**Figure S5.** Methods workflow

**Table S6.** Data sets used in the development of Low Impact Areas.

| **Data Set** | **Units** | **Spatial Resolution** | **Data Year** | **Source** |
| --- | --- | --- | --- | --- |
| Land cover | Land cover type | 10 arc-second | 2015 | ESA Land Cover Climate Change Initiative; Defourney et al. 2017 |
| Global protected areas | IUCN Protection Category | Points and polygon | 2018 | World Database on Protected Areas; UNEP-WCMC & IUCN 2018 |
| Human population density | People/km^2^ (calculated) | 30 arc-second | 2015 | LandScan; Dobson et al. 2000; UT Battelle 2017 |
| Ecoregions | Ecoregion type | Polygon | 2017 | Ecoregions 2017; Dinerstein et al. 2017 |
| Livestock Density | Livestock units/km^2^ (calculated) | 30 arc-second | 2006 | Gridded Livestock of the World v2; Robinson et al. 2014 |
| Global aridity index | Aridity Index | 30 arc-second | 1950-2000 (average) | CGIAR-CSI Global-Aridity and Global PET Database; Zomer et al. 2008; Trabucco & Zomer 2009 |
| Nighttime lights | Radiance | 15 arc-second | 2015 | Nighttime lights version 1. Prepared by Earth Observation Group at NOAA/NCEI using Visible Infrared Imaging Radiometer Suite (VIIRS) Day/Night Band (DNB) data; Elvidge et al. 2017 |
| Forest cover change | Binary (loss & gain) | 30 m | 2000-2015 | Global Forest Watch; Hansen et al. 2013 |
| MODIS Burned Area | Burn date | 500 m | 2000-2015 | MODIS collection 6, MCD64A1 Burned Area; Giglio et al. 2015 |

**Supplementary Text 2 – ‘Very Low Impact Areas’**

Low Impact Areas (LIAs) are created without regard to the presence or absence of roads. However, roads are known to have many negative environmental impacts, particularly the first roads that provide easy human access to a region (Laurance et al. 2009). Roads result in direct habitat destruction, increase rates of illegal logging and poaching, and cause water, air and noise pollution (Spellerberg 1998, Benitez-Lopez et al. 2010). Importantly, roads are also a primary source of habitat fragmentation (Laurance et al. 2009). However, global road data sets are imperfect. The most recent and comprehensive are Open Street Map (OSM) (OSM 2017, Barrington-Leigh and Millard-Ball 2017), gROADS (CIESIN 2013) and GRIPv4 (Meijer et al. 2018). GRIPv4, the newest data set, combined nearly 60 separate data sets on road infrastructure, and provided additional attributes such as road type. Despite this, the data sets vary greatly in data quality between countries and regions of the world. Even in places like the United States, it is not difficult to find gaps or errors. Coverage of the road network can also vary substantially between countries and, in the case of Open Street Map, the density and interests of OSM contributors influences data density and completeness (Girres and Touya 2010, Haklay 2010). For example, we found more roads mapped in popular Tanzanian protected areas than in neighboring rural areas. Therefore, given the uncertainty in quality and completeness of the spatial data, and not wanting to eliminate potential LIAs disproportionally based off data contributors’ interests (e.g. tourism hotspots), we left roads out of the LIA data set, but included them when identifying ‘very low impact areas’ (VLIAs).

We also addressed another important consideration, the application of human population and livestock density data, in the identification of VLIAs. To identify LIA’s, we scaled human and livestock population data to aridity, such that higher densities were required to move a cell from low impact to non-low impact in more humid environments. In the identification of VLIAs, we did not scale by aridity, and kept a consistent threshold of less than one person or livestock unit per km^2^ across the globe.

Therefore, in the identification of VLIAs, we used the same methods as for LIA’s, but did not vary livestock or human population density by aridity, and included all roads from the GRIP v2 data set (Meijer et al. 2018). We used GRIP v4 as the newest and most comprehensive road data set, and excluded any cell where any type of road was present from potential VLIAs.

These two changes substantially expanded the global area of human impact (Figure S5). LIAs represent 56% of the terrestrial, non-permanent ice and snow, whereas VLIAs are only 34%. Large LIAs such as the Congo basin and the Arabian Peninsula shrink and splinter, but much of the speckling of small LIAs also disappears. Indeed, there are fewer patches of very low impact than low impact (a 650% increase vs 1250% increase in patch number from the baseline) (Table S6). Mean patch size is essentially the same between VLIA and LIA, with very low impact patches slightly larger. This is due to the elimination of many small LIAs. Despite mean patch size staying relatively similar between the two methods, core area decreases substantially with VLIAs compared to LIAs (72% to 53%). The differences between the two methods, while substantial, do not alter the ranking of biomes by threat. Tropical dry forests, temperate grasslands and tropical coniferous forests are still the most threatened biomes, all with less than 5% of VLIA remaining and exhibit some of the highest fragmentation rates. Importantly, tropical moist forest is the only biome not cold, dry or high, with some ecoregions having >50% of area in Very Low Impact (Supplementary Figure S7).

| A | 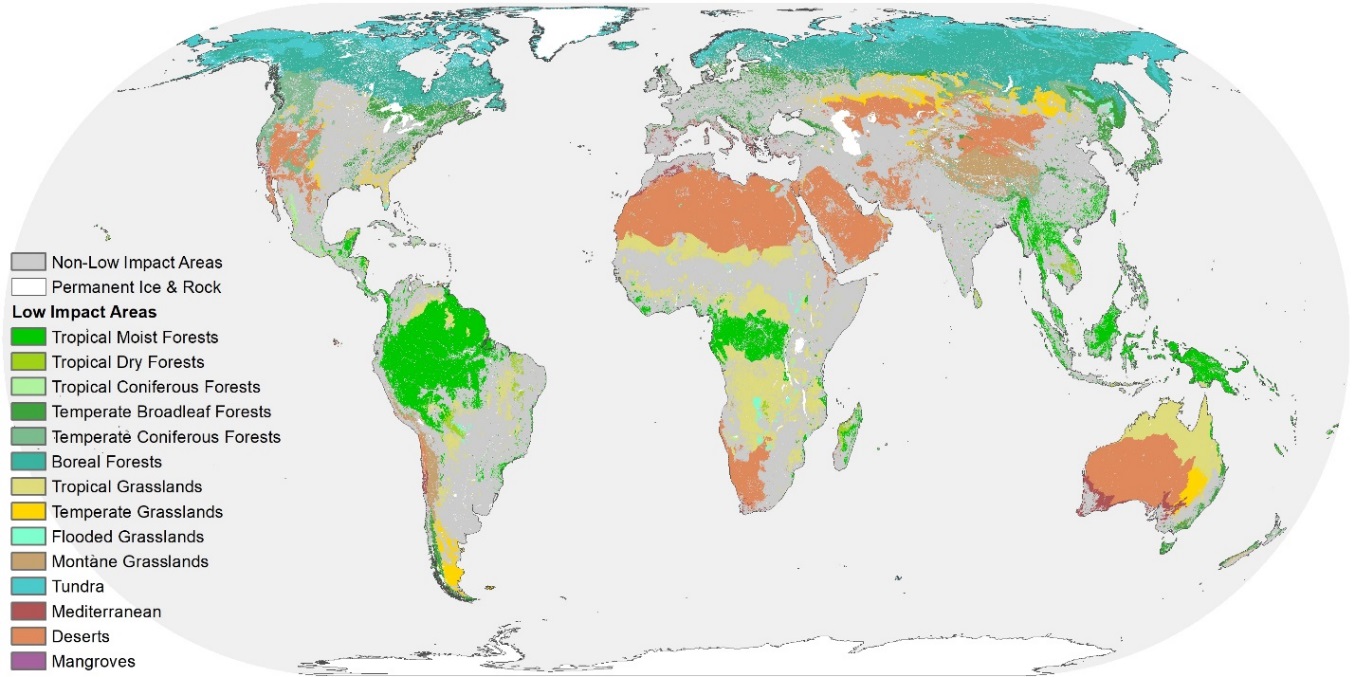 |
| --- | --- |
| B | 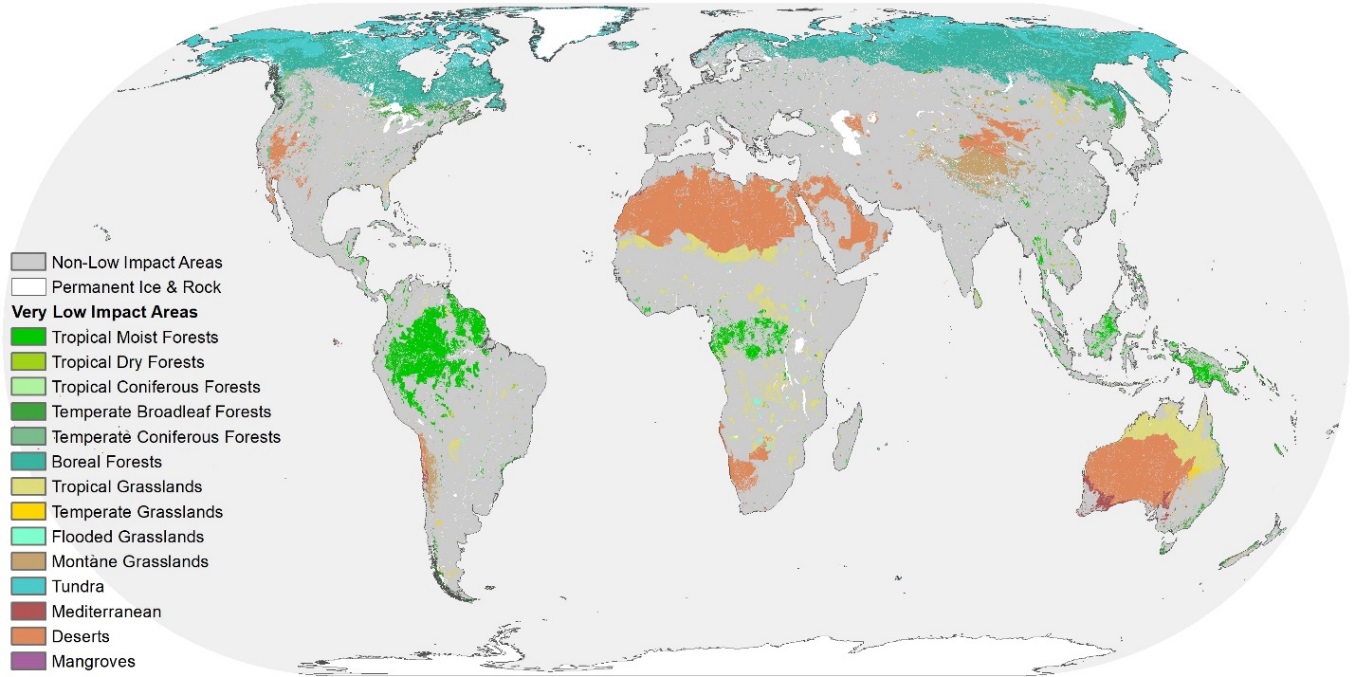 |

**Figure S6.** Maps showing the terrestrial surface of the planet classified as either **A**. Low Impact Areas or **B.** Very Low Impact Areas, color-coded by biome.

**Table S7.** Comparison of key habitat loss and fragmentation statistics between Low Impact Areas and Very Low Impact Areas.

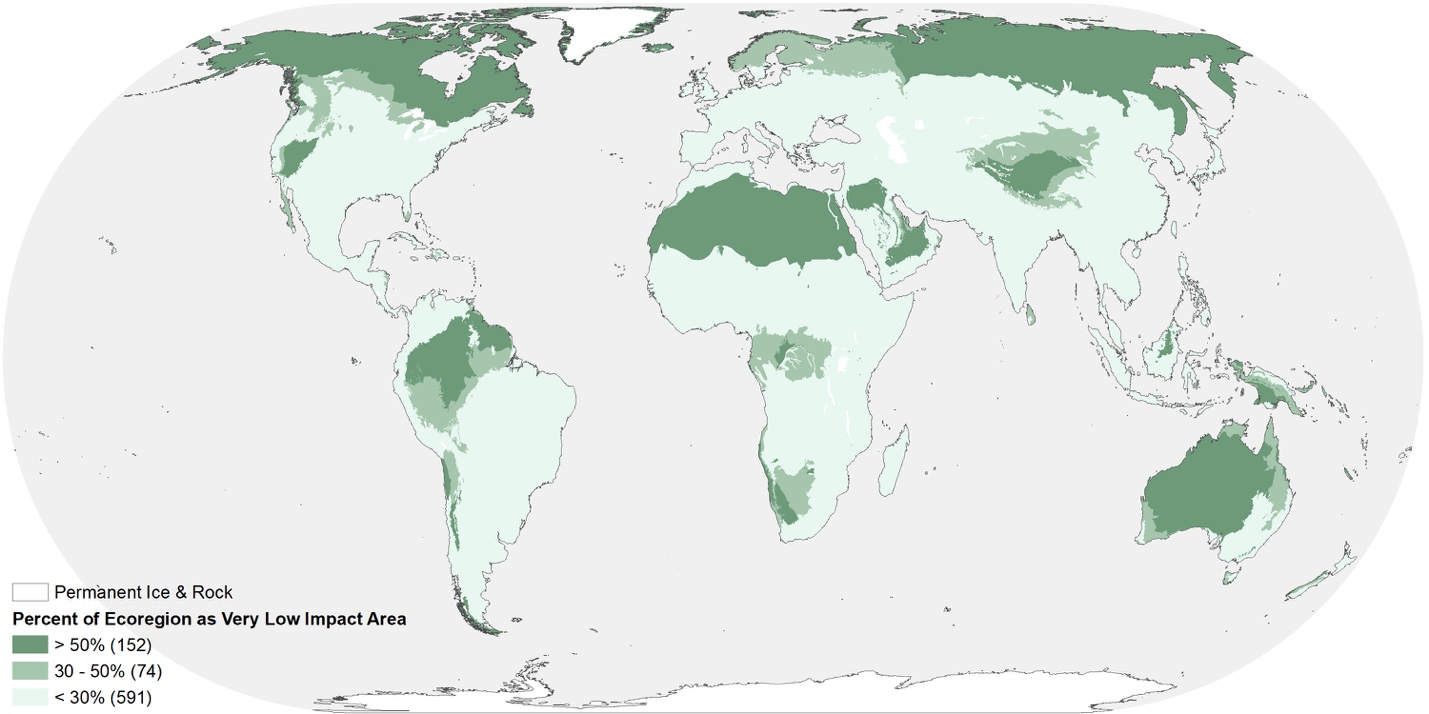


**Figure S7.** Global map highlighting ecoregions that have between 30% and 50% Very Low Impact Area.

**Supplementary Text 3 – Sensitivity of Low Impact Areas to human population and livestock density data sets and scaling**

To test how sensitive the final LIA data set was to decisions about the inclusion of various human impact input data sets, in particular the human population and livestock density data sets and aridity scaling, we conducted several tests.

First, we ran a “leave-one-out” analysis, whereby we left all human stressors in the analysis minus a single stressor, to see how the exclusion of a stressor impacted the final outcome. This showed that the final LIA data set is most sensitive to livestock density, with an additional 11.7% of the world being in a low impact state if it was excluded (Table S8). No other individual stressor had a greater than 4% difference on the final output. Conversely, the LIA data set is least sensitive to nighttime lights, with only an additional 0.6% of the world being in a low impact state with its exclusion. The decision to add back in strict protected areas, to help account for underlying errors in the modelled human population and livestock density data sets, resulted in adding back 1.2% of the terrestrial surface of the world as being in a low impact state. Conversely, our decision to not include roads (of all types) in the standard LIA analysis resulted in an additional 2.3% of the planet as being in a low impact state.

Then, we examined the impact of removing the human population and livestock density data sets completely from the analysis to examine how it would affect fragmentation results. This was termed ‘modified low impact area.’ We compared median distance to edge values across three versions of LIAs, the modified low impact area, the original LIAs where human population and livestock density is scaled by aridity, and the very low impact areas described in Supplementary Text 2 (Supplementary Table S9). Supplementary Figure S8 illustrates the fragmentation and habitat loss of the Sudd wetland in South Sudan across these various LIA versions.

Finally, we reviewed the impact of various scaling factors for human population and livestock density on the extent of LIAs. We used a series of single thresholds for human population and livestock density (the only inputs that used a threshold in our analysis) that matched the same thresholds used in the aridity-scaled analysis (1, 2, 4, 8 and 16 people or livestock units per km^2^). While the final LIA data set using the aridity-scaled human population and livestock unit density resulted in 56.3% of the world in a low impact state, between 40.0% (1 person or livestock unit per km^2^) and 65.7% (16 people or livestock units per km^2^) would have been inferred to be in a low impact state if we used a single threshold for these data sets (Table S10). This is a difference of between -16.3% and +9.4% of the terrestrial surface of the planet and illustrates just how important the chosen threshold is for these data sets. We reiterate that the impacts of both human population and livestock density vary with ecosystem productivity and so we chose to scale these impacts by aridity; such that higher densities were required to move a cell from low impact to non-low impact in more humid environments.

**Table S8.** Sensitivity of the extent of Low Impact Areas to the exclusion (or inclusion in the case of roads) of each human stressor input in the model, using a ‘leave-one-out’ analysis.

|  | Low Impact Area | Change from Low Impact Area |
| --- | --- | --- |
| Original Low Impact Area | 56.3% |  |
| Livestock Unit Density (scaled) | 68.0% | +11.7% |
| Land Cover (Crop & Urban) | 59.9% | +3.7% |
| Human Population Density (scaled) | 59.2% | +2.9% |
| Forest Cover Change | 57.6% | +1.4% |
| Nighttime Lights | 56.9% | +0.6% |
| Strict Protected Areas | 55.1% | -1.2% |
| Roads (included) | 54.0% | -2.3% |

**Table S9.** Median distance to edge values for the world’s 14 biomes and the percent Low Impact Area within them. ‘Modified LIA’ represents low impact areas without the inclusion of human population or livestock density data sets, and is 75% of the world. LIAs are the standard Low Impact Areas as described in the text, and is 56% of the world. Very Low Impact Areas are LIA’s with no scaling of human population or livestock density by aridity (i.e. set at one), and where roads are included as a human impact variable, and is only 34% of the world.

**
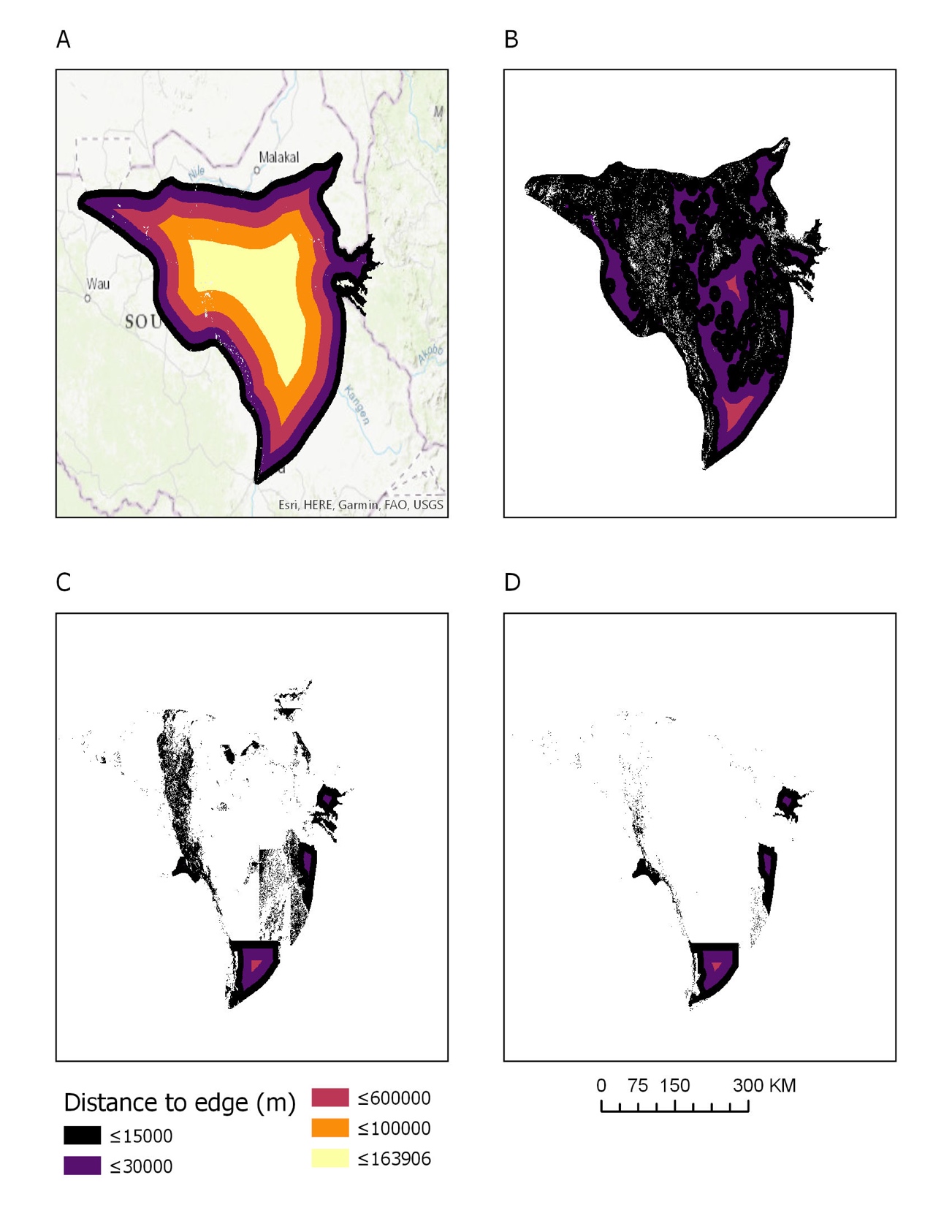
**

**Figure S8.** Distance to edge figures for the Sudd flooded grassland ecoregion in South Sudan, Africa. **A.** Distance to biome edge. Permanent water, from the land cover data, is shown as empty cells. **B.** Distance to biome edge or modified non-Low Impact Area cell (the modified low impact areas are where human and livestock densities are not used in delineating Low Impact Areas). **C.** Distance to biome edge or non-Low Impact Area cell for the standard Low Impact Areas (where human and livestock densities are scaled by aridity). **D.** Distance to biome edge or non-Low Impact Area cell for the Very Low Impact Areas (with roads included, and human and livestock densities not scaled by aridity but set at one).

**Table S10.** Sensitivity of the extent of Low Impact Areas to the use of aridity-scaled thresholds for human population and livestock density data. The change in the extent of Low Impact Area from aridity-scaled thresholds to various single, global thresholds is shown.

|  | Low Impact Area | Change from Low Impact Area |
| --- | --- | --- |
| Original Low Impact Area  (Aridity-scaled) | 56.3% |  |
| 1 Person or Livestock Unit/km^2^ | 40.0% | -16.3% |
| 2 People or Livestock Units/km^2^ | 46.3% | -9.9% |
| 4 People or Livestock Units/km^2^ | 52.8% | -3.5% |
| 8 People or Livestock Units/km^2^ | 59.3% | +3.0% |
| 16 People or Livestock Units/km^2^ | 65.7% | +9.4% |

**Table S11.** Comparison in fragmentation statistics using either 4-cell (connection only where edges meet) or 8-cell (connection at edges and diagonal) adjacency rules. 4-cell adjacency is used in the paper.

| Biome | **8 cell: # of historic patches** | 4 cell: # of historic patches | **8 cell: # of LIA patches** | 4 cell: # of LIA patches | **8 cell: % Increase in patch #** | 4 cell: % Increase in patch # | **8 cell: % Decrease in mean patch size** | 4 cell: % Decrease in mean patch size | **8 cell: % Decrease in core area** | 4 cell: % Decrease in core area |
| --- | --- | --- | --- | --- | --- | --- | --- | --- | --- | --- |
| Globe | **30,460** | 73,374 | **508,833** | 993,656 | **1570%** | 1254% | **96.6%** | 95.8% | **53.4%** | 53.4% |
| Tropical dry forests | **585** | 1,317 | **16,024** | 27,375 | **2639%** | 1979% | **99.4%** | 99.2% | **89.6%** | 89.6% |
| Temperate grasslands | **835** | 2,340 | **85,379** | 146,608 | **10125%** | 6165% | **99.8%** | 99.6% | **84.3%** | 84.3% |
| Tropical coniferous forests | **530** | 767 | **4,554** | 8,345 | **759%** | 988% | **96.7%** | 97.4% | **83.6%** | 82.5% |
| Mediterranean | **727** | 1,354 | **21,979** | 38,478 | **2923%** | 2742% | **99.0%** | 99.0% | **82.5%** | 81.0% |
| Temperate broadleaf forests | **3,151** | 8,312 | **112,624** | 208,314 | **3474%** | 2406% | **99.1%** | 98.8% | **81.0%** | 83.6% |
| Flooded grasslands | **945** | 2,375 | **7,725** | 13,450 | **717%** | 466% | **95.5%** | 93.5% | **72.3%** | 72.3% |
| Tropical grasslands | **1,986** | 5,108 | **91,058** | 171,346 | **4485%** | 3254% | **99.1%** | 98.7% | **70.8%** | 65.7% |
| Mangroves | **3,013** | 5,345 | **6,081** | 10,188 | **102%** | 91% | **77.3%** | 76.0% | **65.7%** | 70.8% |
| Tropical broadleaf forests | **3,981** | 9,153 | **99,159** | 189,727 | **2391%** | 1973% | **97.9%** | 97.4% | **56.7%** | 56.7% |
| Montane grasslands | **1,632** | 5,105 | **12,920** | 29,278 | **692%** | 474% | **92.6%** | 89.8% | **52.4%** | 52.5% |
| Temperate coniferous forests | **1,245** | 2,662 | **16,337** | 39,369 | **1212%** | 1379% | **94.8%** | 95.4% | **49.4%** | 49.5% |
| Deserts | **1,166** | 2,731 | **30,730** | 59,457 | **2536%** | 2077% | **97.3%** | 96.8% | **34.2%** | 34.2% |
| Boreal forests | **3,672** | 13,886 | **15,148** | 59,801 | **313%** | 331% | **77.6%** | 78.5% | **18.7%** | 18.7% |
| Tundra | **17,075** | 34,390 | **17,850** | 36,159 | **5%** | 5% | **5.0%** | 5.6% | **1.9%** | 1.9% |

**References from Supplemental Information**

Barrington-Leigh, C., & Millard-Ball, A. The world’s user-generated road map is more than 80% complete. *PloS one* ***12***, p.e0180698 (2017).

Benítez-López, A., Alkemade, R. & Verweij, P.A. The impacts of roads and other infrastructure on mammal and bird populations: a meta-analysis. *Biological Conservation* **143** 1307–16 (2010).

Center for International Earth Science Information Network—CIESIN—Columbia University and Information Technology Outreach Services—ITOS—University of Georgia *Global Roads Open Access Data Set, Version 1* (Palisades, NY: NASA Socioeconomic Data and Applications Center) (2013).

Defourny, P., Bontemps, S., Brockmann, C., Boettcher, M., Wevers, J. & Kirches, G. Land Cover CCI: Product User Guide Version 2.0 (2017).

Dobson, J.E., Bright, E.A., Coleman, P.R., Durfee, R.C. & Worley, B.A. LandScan: a global population database for estimating populations at risk. *Photogrammetric engineering and remote sensing* **66**, 849-857 (2000).

Ellis, E.C. & Ramankutty, N. Putting people in the map: anthropogenic biomes of the world. *Frontiers in Ecology and the Environment* **6**, 439-447 (2008).

Ellis, E.C., Klein Goldewijk, K., Siebert, S., Lightman, D. & Ramankutty, N. Anthropogenic transformation of the biomes, 1700 to 2000. *Global ecology and biogeography* **19**, 589-606 (2010).

Elvidge, C.D., Baugh, K., Zhizhin, M., Hsu, F.C. & Ghosh, T. VIIRS night-time lights. *International Journal of Remote Sensing* **38**, 5860-5879 (2017).

Giglio, L., Schroeder, W. & Justice, C.O. The collection 6 MODIS active fire detection algorithm and fire products. *Remote Sensing of Environment* **178**, 31-41 (2016).

Girres, J.F. & Touya, G. Quality assessment of the French OpenStreetMap dataset. *Transactions in GIS* ***14,*** 435-459 (2010).

Haklay, M. How good is volunteered geographical Information? A comparative study of OpenStreetMap and Ordnance Survey datasets. *Environment and Planning B* **37**, 682–703 (2010).

Hannah, L., Lohse, D., Hutchinson, C., Carr, J.L. & Lankerani, A. A preliminary inventory of human disturbance of world ecosystems. *Ambio*, 246-250 (1994).

Hansen, M.C. *et al.* High-resolution global maps of 21st-century forest cover change. *Science* **342**, 850-853 (2013).

Laurance, W.F., Goosem, M. & Laurance, S.G., Impacts of roads and linear clearings on tropical forests. *Trends in ecology & evolution* ***24,*** 659-669 (2009).

Kennedy, C., Oakleaf, J.R., Theobald, D.M., Baruch-Mordo, S. & Kisecker, J. Managing the Middle: A shift in conservation priorities based on the global human modification gradient. *Global Change Biology* **25**, 811-826 (2019).

McCloskey, J.M. & Spalding, H. A reconnaissance-level inventory of the amount of wilderness remaining in the world. *Ambio*, 221-227 (1989).

Meijer, J.R., Huijbregts, M.A., Schotten, K.C. & Schipper, A.M., 2018. Global patterns of current and future road infrastructure. *Environmental Research Letters* ***13***, p.064006.

OSM. *OpenStreetMap, the Free Wiki World Map* (West Midlands: OSM) 2009. [www.openstreetmap.org](http://www.openstreetmap.org)

Robinson, T.P *et al.* Mapping the global distribution of livestock. *PloS one* ***9***, p.e96084 (2014).

Sanderson, E.W., Jaiteh, M., Levy, M.A., Redford, K.H., Wannebo, A.V. & Woolmer, G. The human footprint and the last of the wild: the human footprint is a global map of human influence on the land surface, which suggests that human beings are stewards of nature, whether we like it or not. *AIBS Bulletin* **52**, 891-904 (2002).

Spellerberg, I.A.N.. Ecological effects of roads and traffic: a literature review. *Global Ecology & Biogeography Letters* ***7***, 317-333 (1998).

Trabucco, A. & Zomer, R.J. Global aridity index (global-aridity) and global potential evapo-transpiration (global-PET) geospatial database. *CGIAR Consortium for Spatial Information* (2009).

UNEP-WCMC & IUCN, Protected Planet: The World Database on Protected Areas. (UNEP-WCMC and IUCN, Cambridge, 2018). Downloaded July 2018.

UNEP-WCMC. Assessing forest integrity and naturalness in relation to biodiversity: Forest Resource Assessment – WP54 (2000). <http://www.fao.org/3/ad654e/ad654e00.htm>

Venter, O. et al. Data from: Global terrestrial Human Footprint maps for 1993 and 2009. Dryad Digital Repository (2016a). <https://doi.org/10.5061/dryad.052q5.2>

Venter, O. *et al.* Sixteen years of change in the global terrestrial human footprint and implications for biodiversity conservation. *Nature Communications* **7**, 12558 (2016b).

Zomer, R.J., Trabucco, A., Bossio, D.A. & Verchot, L.V. Climate change mitigation: A spatial analysis of global land suitability for clean development mechanism afforestation and reforestation. *Agriculture, ecosystems & environment* **126**, 67-80 (2008).
